# Imaging-Based In Situ Analysis of 5-Methylcytosine at Low Repetitive Single Gene Loci with Transcription-Activator-Like Effector Probes

**DOI:** 10.1021/acschembio.2c00857

**Published:** 2023-01-24

**Authors:** Anne Jung, Álvaro Munõz-López, Benjamin C. Buchmuller, Sudakshina Banerjee, Daniel Summerer

**Affiliations:** † Faculty of Chemistry and Chemical Biology, TU Dortmund University, Otto-Hahn-Str. 6, 44227 Dortmund, Germany; ‡ International Max Planck Research School of Living Matter, Otto-Hahn-Str. 11, 44227 Dortmund, Germany

## Abstract

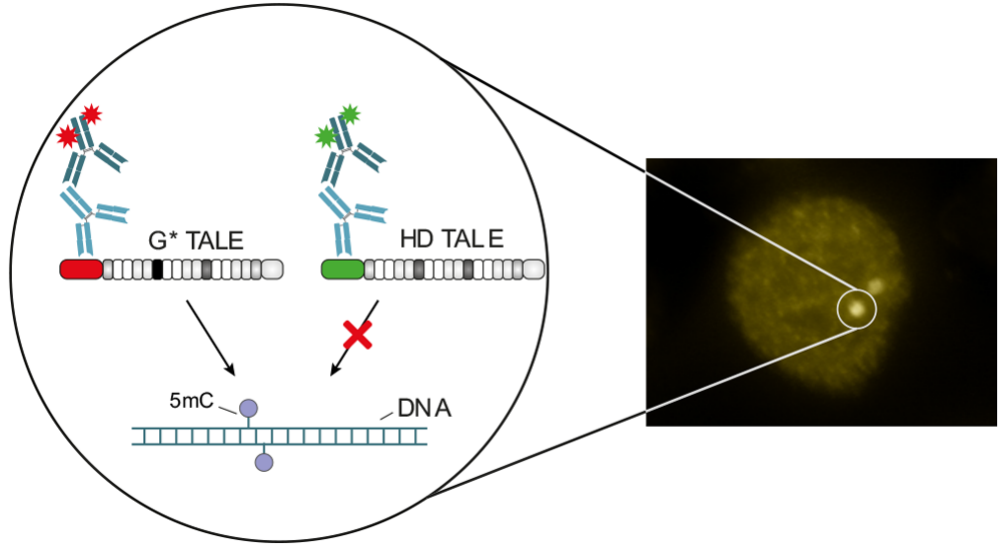

Transcription-activator-like effectors (TALEs) are programmable DNA binding proteins that can be used for sequence-specific, imaging-based analysis of cellular 5-methylcytosine. However, this has so far been limited to highly repetitive satellite DNA. To expand this approach to the analysis of coding single gene loci, we here explore a number of signal amplification strategies for increasing imaging sensitivity with TALEs. We develop a straightforward amplification protocol and employ it to target the MUC4 gene, which features only a small cluster of repeat sequences. This offers high sensitivity imaging of MUC4, and in costaining experiments with pairs of one TALE selective for unmethylated cytosine and one universal control TALE enables analyzing methylation changes in the target independently of changes in target accessibility. These advancements offer prospects for 5-methylcytosine analysis at coding, nonrepetitive gene loci by the use of designed TALE probe collections.

5-Methylcytosine (5mC, Figure 1a) is the main epigenetic modification of mammalian genomes and plays essential roles in the regulation of transcription, cell differentiation and development.^1^ 5mC is written onto CpG dyads by DNA methyltransferases (DNMT), and perturbations of this process are early events in the formation of cancer.^2^ Locus-specific 5mC functions are typically studied by its sequencing-based analysis in purified DNA, and correlations with other local chromatin features.^3^ Recently, strategies for the imaging-based *in situ* analysis of cellular 5mC in repeat DNA have been introduced and promise to enable direct correlations at user-defined sequences of single cells.^4,5^ For example, cellular 5mC has been imaged using *in situ* hybridization (FISH) probes that can cross-link to 5mC,^6^ or using combinations of a methyl-CpG-binding domain protein and a programmable DNA-binding protein in fluorescence complementation designs.^7^ We recently reported an approach solely based on fluorescently labeled transcription-activator-like effector (TALE) proteins^8−10^ that serve as 5mC-sensitive imaging probes.^11^ TALEs bind one strand of double stranded DNA via a programmable domain of concatenated repeats, each recognizing one nucleobase via a so-called repeat variable diresidue (RVD, Figure 1b), thus offering DNA sequence recognition with nucleotide resolution.^12,13^ A number of repeats have been engineered to exhibit selectivity for epigenetically modified cytosines, or to show universal binding of nucleobases.^14−17^ Our imaging approach relied on costains of fixed mammalian cells with two different TALEs being fused to two different fluorescent proteins. One TALE thereby binds a target CpG cytosine with an HD RVD that is blocked by 5mC (Figure 1c), the other TALE binds the same cytosine in the target with the RVD G* that universally binds to any nucleobase with similar affinities (including C and 5mC; Figure 1d).^18^

**Figure 1:**
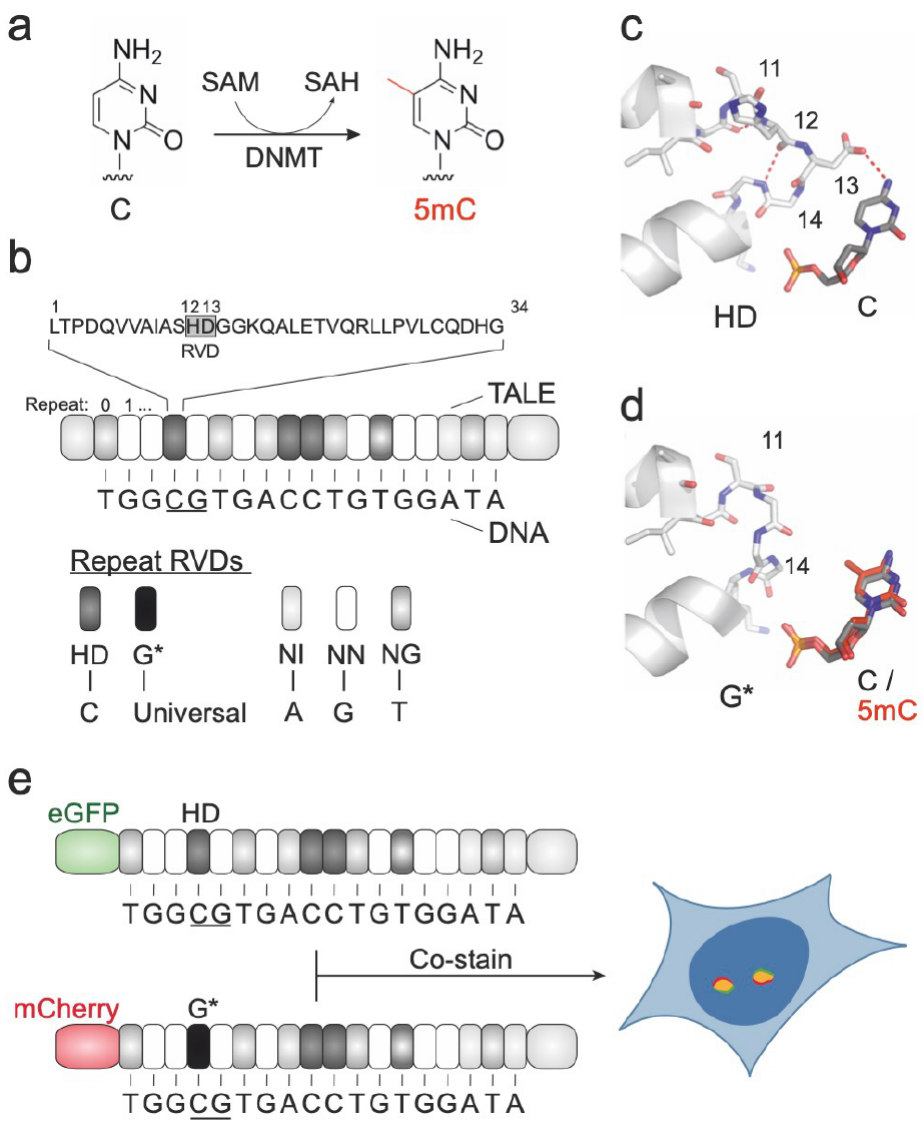
DNA recognition and cell staining by TALEs. a) Cytosine 5-methylation. SAM: S-adenosylmethionine; SAH: S-adenosylhomocysteine. b) TALE features. Sequence of one repeat is shown on top with RVD in gray box. RVD selectivities are shown below. * = deletion. c) Crystal structure of RVD HD bound to C.^13^ d) Model of RVD G* bound to C or 5mC.^15^ e) Co-staining approach with C-selective TALE and universal control TALE.

This second TALE served as internal control that marks the target loci and because of its 5mC insensitivity is expected to be affected solely by differences in accessibility of the target (e.g., due to 5mC-induced chromatin condensation), which offers dissecting such differences from differences in 5mC itself. We previously applied this approach to pericentromeric SATIII DNA, a class of clustered repeats that is the origin of nuclear stress bodies (nSB) and shows perturbed methylation profiles in a number of cancer cells.^19^ However, SATIII DNA is a straightforward imaging target, since it constitutes >1% of the genome^20^ and thus provides excellent signal noise ratios. This current restriction to repeat DNA targets of our (and other approaches) severely limits the application scope by preventing the in situ methylation analysis of coding single genes.

To increase the sensitivity of our approach and enable single gene analyses, we explored a number of signal amplification strategies. Starting with a reference target with known staining behavior, we employed a SATIII-directed TALE that binds a 18mer sequence with a single CpG in the 5′-region (TALE_SATIII, Figure 2a). This TALE has a variety of highly repetitive target sequence clusters (with a maximum of 28k repeats in chromosome 9,^21^ see SI table S5 for full analysis). For signal amplification, we first focused on TALE-mCherry fusions and used other fluorophores in a similar spectral range for amplification. Specifically, we constructed and expressed universally binding G* repeat versions of TALE_SATIII bearing as N-terminal tag either one or three mCherry proteins, 20 or 30 concatenated FLAG tag copies, or a Sun-Tag (bearing 24 copies of a GCN4 peptide;^22^Figure 2 a). We thereby tested a number of different nanobody or antibody signaling and signal amplification strategies for the employed tags (Figure 2a). We initially stained U2OS cells with TALE_SATIII bearing 1x or 3x mCherry fusions, but did not observe significant differences between the two TALEs (Figure 2b, c). Moreover, subsequent staining of the cells with an mCherry-selective nanobody-ATTO594 conjugate (“RFP-booster”) did not afford significantly enhanced fluorescence for either TALE (Figure 2b, c).

**Figure 2:**
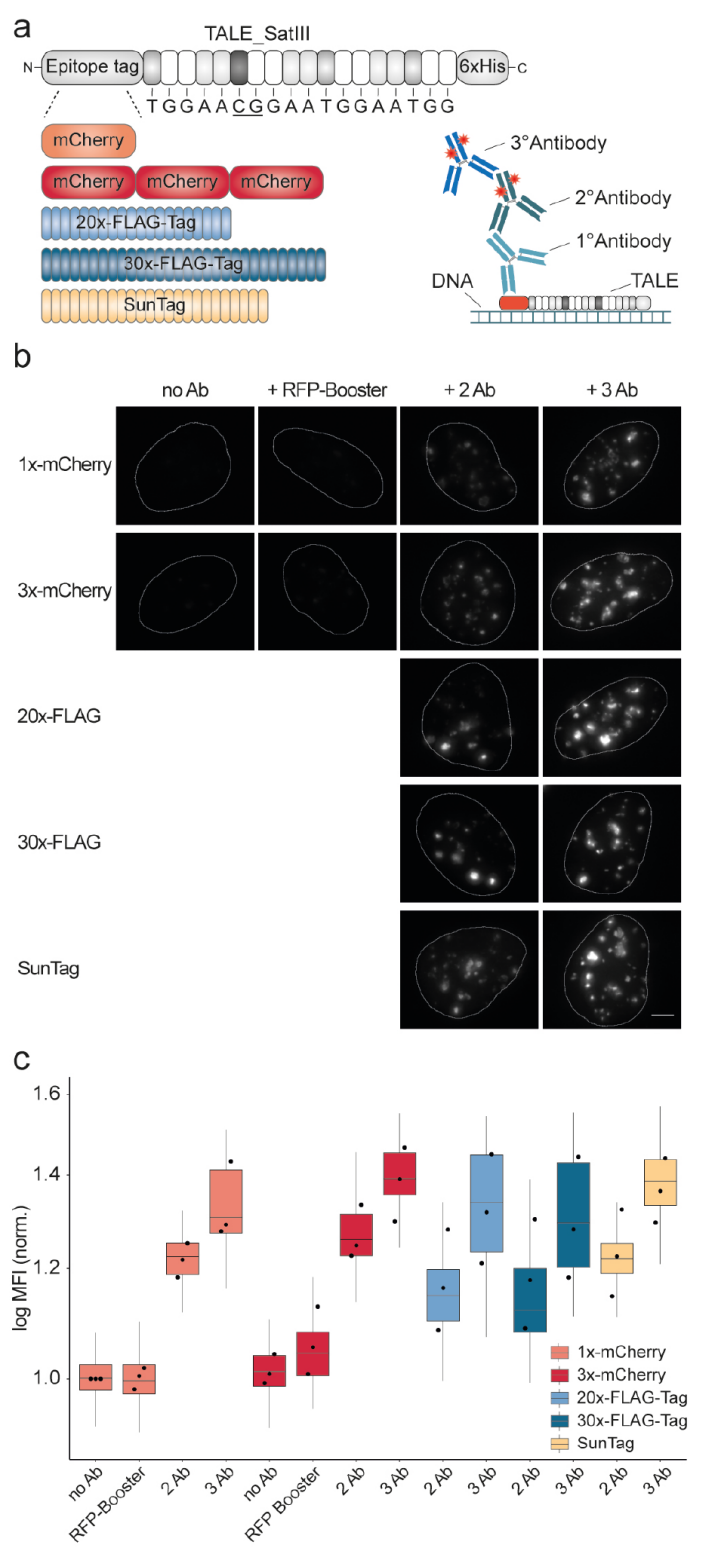
Fluorescence signal amplification. a) Left: TALE structure with different N-terminal epitope tags. Right: TALE epitope tag immunostaining with fluorescently labeled antibodies. b) TALE_SATIII staining with different epitope tag and immunostaining combinations. Representative images of foci in nuclei of U2OS cells. All images were acquired under identical imaging conditions set at low light intensity and short exposure times to correctly visualize SATIII at the highest signal values. No Ab: No antibody, RFP-Booster: RFP-Booster ATTO594, 2 Ab: Primary antibody against epitope tag and secondary Alexa Fluor plus 594 labeled antibody, 3 Ab: Primary antibody against epitope tag, secondary Alexa Fluor plus 594 labeled antibody and tertiary Alexa Fluor plus 594 labeled antibody. Scale bar: 5 μm. c) Mean fluorescence intensities of foci from signal amplification conditions shown in Figure 2b normalized to 1x-mCherry no Ab condition. Dots represent normalized mean values of each experiment with *N* = 3 totaling 2802 cells and 44038 foci.

We next evaluated combinations of an anti-mCherry antibody and a secondary antibody labeled with Alexa Fluor plus 594 (Figure 2a). This led to significant fluorescence enhancements for the 1x and 3x-mCherry constructs, with slightly higher signals for the 3x-mCherry constructs (4.1- and 4.8-fold increase of absolute fluorescence intensity; Figure 2b, c (Figure 2c shows log. normalized fluorescence intensities)). Moreover, subsequent staining with an additional tertiary antibody labeled with the same fluorophore further increased the absolute fluorescence 2.1-fold for both, the 1x-mCherry constructs and 3x-mCherry constructs (Figure 2b, c; i.e., 8.8-fold and 10.3-fold from no to three antibody amplification). For TALE constructs bearing 20x or 30x FLAG tags stained with a primary anti-FLAG antibody and Alexa Fluor plus 594-labeled secondary antibody, we observed about 3.5-fold higher absolute fluorescence signals compared to the tested mCherry constructs alone, but the signals were slightly lower than for the mCherry constructs in combination with double antibody staining (Figure 2 b, c). The signals thereby did not differ between the 20x and 30x FLAG constructs. Use of a tertiary labeled antibody for the FLAG-constructs afforded similar signals as the same strategy for the mCherry constructs (Figure 2b, c). Finally, staining with SunTag-labeled TALEs and two or three antibodies afforded similar signals as compared to the mCherry-based stains (Figure 2b, c).

We next designed a number of TALE probes targeting sequences that occur multiple times at the MUC4 locus. This locus encodes the high molecular weight transmembrane mucin MUC4, which shows aberrant expression in a variety of carcinomas.^23^ MUC4 is mainly expressed in airway epithelial cells for protection and lubrication, and plays important roles in cell signaling, proliferation and differentiation.^24^ Mucins contain heavily glycosylated central domains, composed of a variable number of tandem repeats. Due to the repetitive nature of the MUC4 locus, it has previously been a target for live and fixed cell imaging using programmable DNA binding domains such as TALEs and CRISPRS-dCas9 as probes.^25,26^ We designed mCherry-fused TALEs M1–3 with varying numbers (148 to a minimum of 32) of theoretical target sequences at the locus and conducted imaging experiments with U2OS cells (note that the latest long read-based genome assembly T2T-CHM13v2.0^21^ afforded an increased number of MUC4 repeats, which affects the actual target number of imaging probes also of previous publications, SI Table S6). We observed an expected staining pattern for a single locus in a diploid cell with two foci in the majority of cells^25−28^ for all three TALEs (Figure 3b and SI; dividing cells showed an expected number of four foci, SI Figure S1). Importantly, costains with all possible combinations of the three TALEs afforded colocalized foci in all cases, indicating selective staining of the MUC4 locus (SI Figure S2).

**Figure 3:**
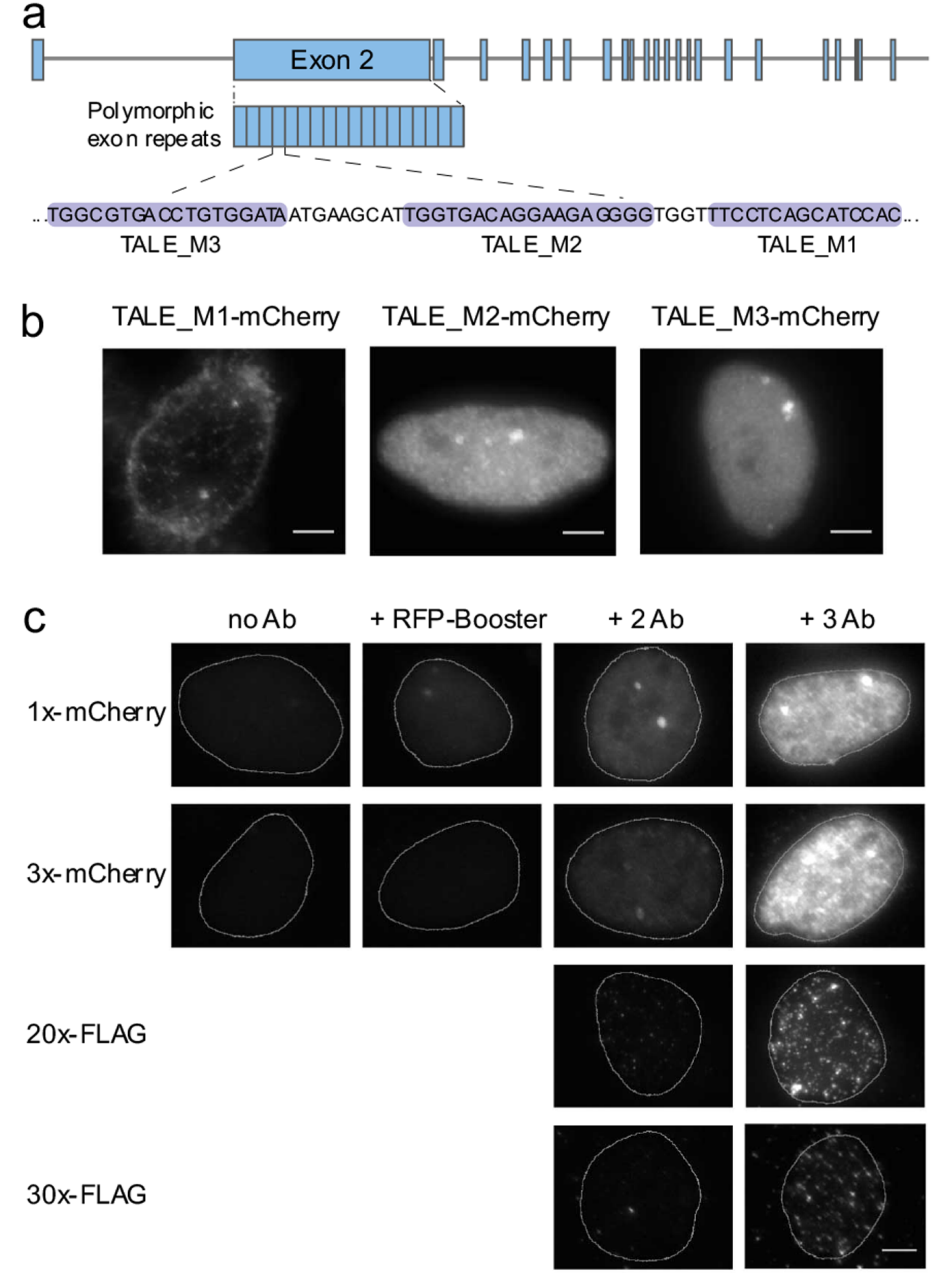
Staining of low repetitive TALE target sequences at the MUC4 locus. a) TALE target sequences (purple) in polymorphic exon 2 repeats of MUC4 locus. b) Images of U2OS cells stained with TALEs with 148, 79, and 32 target sequences showing MUC4-characteristic foci (stains were conducted with TALE_M1–3x-mCherry, TALE_M2–1x-mCherry or TALE_M3–1x-mCherry, followed by immunostaining with a primary antibody against mCherry and a secondary Alexa Fluor plus 594 labeled antibody. Scale bars: 5 μm). c) Staining experiments using TALE_M3 bearing different epitope tags in combination with different immunostainings. Representative images of foci in nucleus of U2OS cells are shown. All fluorescence images were acquired under identical imaging conditions. No Ab: No antibody, RFP-Booster: RFP-Booster ATTO594, 2 Ab: Primary antibody against epitope tag and secondary Alexa Fluor plus 594 labeled antibody, 3 Ab: Primary antibody against epitope tag, secondary Alexa Fluor plus 594 labeled antibody and tertiary Alexa Fluor plus 594 labeled antibody. Scale bar: 5 μm.

Because of its ability to visualize a target with a particularly low number of repeats, we selected TALE_M3 for further studies. Since this target requires far higher sensitivity and lower background as highly repetitive SATIII DNA, we re-evaluated the most successful signal amplification strategies identified before to MUC4 staining with this TALE (we did not include the SunTag strategy, since we noticed an off-target staining of centriolin with a MUC4-like staining pattern by the employed antibody, complicating its application in this context, SI Figure S3). Similar to the SATIII signal amplification, the RFP Booster did not afford satisfactory signals (Figure 3c and SI Figure S4). However, unlike for SATIII, we observed weaker signals for 3x mCherry compared to 1x mCherry stainings with two antibodies. Moreover, the use of three antibody-amplification led to a granular nucleus staining for both mCherry TALE_M3 constructs. This high background severely complicated selective analysis of MUC4 foci (Figure 3c and SI Figure S4). Surprisingly, we also observed lower signals for both FLAG constructs in combination with two antibody-staining than for the 1x mCherry construct. In addition, the use of three antibody-amplification again led to a granular nucleus background staining (Figure 3c). We therefore ultimately selected the 1x mCherry two-antibody amplification strategy for later experiments, since this afforded the highest signal noise ratio and the most homogeneous staining patterns (Figure 3c and SI Figure S4).

We next aimed to visualize 5mC differences in MUC4 target sequence by using our costaining approach with HD and G* version of TALE_M3. We designed both mCherry and eGFP versions of TALE_M3 bearing either an HD or G* repeat opposite the C nucleobase of the single CpG dyad in the target sequence (Figure 3a). For signal amplification of eGFP TALEs, we employed the analogous strategy as for mCherry, i.e. 1x eGFP and two antibody staining, the second antibody being labeled with Alexa Fluor 488 dyes. This strategy led to very similar staining patterns and signal noise ratios as obtained for mCherry (SI Figure S5). All employed TALEs thereby exhibited clear colocalization, indicating selective recognition of the MUC4 target sequence, despite the differences in repeats and fluorophores (Figure 4c). As target cells for visualization of 5mC differences, we employed HCT116 colon cancer cells either as wild type or bearing a double knockout of DNMT1 and DNMT3b (DKO). The latter cells exhibit <5% of the global 5mC level found in wild type HCT116 cells.^29^ Methylated-DNA-immunoprecipitation (MeDIP) experiments followed by qPCR analyses revealed a relatively small (<2-fold) 5mC difference for SATIII DNA, but a high 11.5-fold 5mC difference for the MUC4 locus between the two cell lines (Figure 4a; see Figure S12 for bisulfite sequencing data). We first conducted costains of both cell lines with MUC4-directed TALE_M3 pairs being either both G* TALEs, or the mCherry version being a G* TALE and the eGFP version being an HD TALE (Figure 4c). We recorded signals for foci showing both mCherry and eGFP fluorescence, and subtracted the extracellular background signal. For a facile comparison, we normalized for each TALE the signals to the mean fluorescence of the HCT116 DKO foci. Both G* TALEs in the former costain did not differ in signals between the two cell types (Figure 4d).

**Figure 4:**
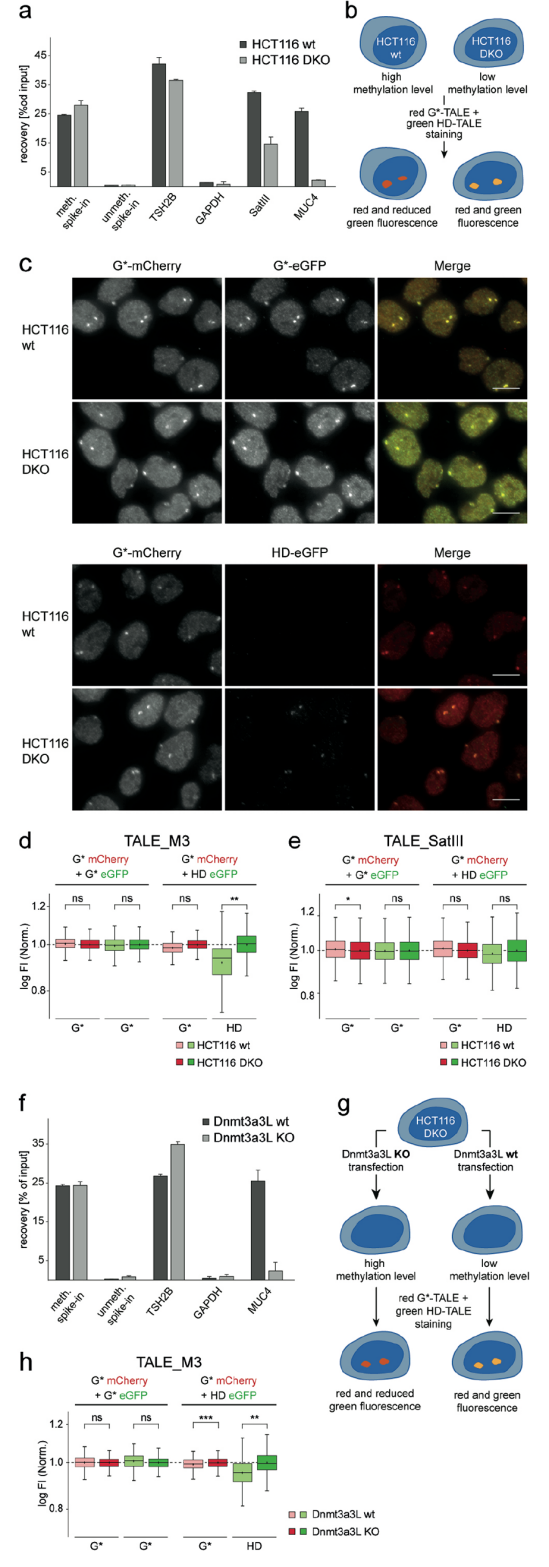
Imaging-based in situ analysis of 5mC by staining with TALE probes. a) MeDIP analysis of wt and DKO HCT116 cells. Shown are relative recoveries of target DNA from both cell types assessed by real-time quantitative PCR (qPCR). Results show means ± SD of technical MeDIP duplicates and qPCR triplicates. Shown are results for methylated and unmethylated spike in controls, for an endogenous control locus with high (TSH2B) and low (GAPDH) 5mC level, and for the SATIII and MUC4 target loci. b) Cartoon of TALE costaining experiment of HCT116 wt and DKO cells of Figure 4d-e, exemplary shown for red G* TALE and green HD TALE. c) Co-stains of HCT116 cells with TALE_M3 1x-mCherry and TALE_M3–1x-eGFP bearing either HD or G* repeats opposite the target CpG. Cells were stained with primary anti-mCherry and anti-eGFP antibodies and secondary Alexa Fluor plus 594 labeled and Alexa Fluor 488 labeled antibodies. Fluorescence images were acquired under identical imaging conditions for each channel. Scale bars: 10 μm. d-e) Fluorescence signal intensities (FI) of foci from HCT116 wt and DKO cells costained with G* and HD TALE_M3 (d) or TALE_SATIII versions (e) fused to mCherry or eGFP and immunostained with primary anti mCherry, secondary Alexa Fluor Plus 594, primary anti-eGFP and secondary Alexa Fluor 488. For each TALE, log FI of each focus is normalized to the mean of log FI of all foci from HCT116 DKO cells. *N* = 4 experiments totaling >2000 foci per condition (d), and >7000 foci per condition (e). *P* < 0.1*, *P* < 0.01**(Student’s *t* test). f) Same as Figure 4a with DNA samples from HCT116 DKO cells transfected with either catalytically active Dnmt3a3L or inactive Dnmt3a3L E756A. g) Cartoon of TALE costaining experiment with HCT116 DKO cells transfected with Dnmt3a3L or inactive E756A mutant to obtain cells with high and low methylation level, respectively. TALE staining is exemplary shown for red G* TALE and green HD TALE. h) Same as Figure d-e with foci from HCT116 DKO cells transfected with active or inactive Dnmt3a3L. *N* = 3 experiments totaling >1300 foci per condition. *P* < 0.01**, *P* < 0.001*** (Student’s *t* test). Further statistics in SI.

In contrast, in the latter costain, the HD TALE showed a significantly lower signal in the wt cells, whereas the G* TALE again did not differ between the two cell types (Figure 4d). We noticed that mCherry TALEs generally did not strongly differ between the two cell types even as HD version (SI Figure S8). We thus used mCherry TALEs only as marker TALEs in the present study, and not as internal controls for chromatin accessibility as in our previous study. Nevertheless, our comparative costain with the eGFP G* TALE not showing a decreased signal in wt cells indicates a selective response of the eGFP HD TALE to 5mC differences itself, rather than differences in target accessibility (Figure 4d). This enables the use of the G* TALE as external control in comparative stainings. Interestingly, we observed a response of the HD TALE also in the nuclear background, suggesting 5mC selectivity also in respect to off-target sequences (see SI Figure S9 for further analyses). We further conducted analogous experiments with the corresponding TALE_SATIII pair. Here, none of the TALEs exhibited marked differences between the two cell lines, in agreement with the low 5mC differences observed in MeDIP analyses (Figure 4e and a).

To further corroborate that the observed responses in our TALE staining were reporting 5mC differences in the MUC4 target sequences, we conducted a costaining experiment with alternative 5mC perturbation. We transfected HCT116 DKO cells with plasmids encoding either a catalytically active or inactive DNMT3a3L construct. This construct allows effective global de novo methylation, and thus rescue of depleted 5mC in DKO cells (Figure 4g).^30^ Indeed, DKO cells transfected with active DNMT3a3L showed very similar 5mC levels as HCT116 wt cells in MeDIP analyses, whereas the inactive DNTM3a3L cells showed a DKO-like level (Figure 4f and a). We observed highly similar staining results with these cells as observed in the wt versus DKO comparison before, confirming a selective reporting of 5mC differences by our TALE costain (Figure 4h and d).

In summary, we report *in situ* analysis of 5mC in user-defined, low repetitive DNA sequences of cells by the use of fluorescent TALE probes. Compared to our previous approach that was limited to highly repetitive SATIII DNA with many thousand genomic copies, the development of an effective signal amplification protocol allowed us to image only 32 theoretical target sequences at the MUC4 locus with a high signal noise ratio. Co-staining experiments with C-selective and universal marker TALEs and comparison with MeDIP data indicate the ability of our approach to report 5mC differences at one CpG in the target sequences independently of differences in target accessibility between cells with different methylation levels. The observed sensitivity for such a small number of target sequences now offers perspectives for 5mC analysis at complex, nonrepetitive single loci by the use of specifically designed TALE probe collections.

## Supporting Information Available

Experimental protocols; Tables S1–S2, oligonucleotide sequences; Table S3, TALE assembly; Table S4, statistical data of quantification shown in Figure 2c; Tables S5–S6, theoretical repeats of sequences in SatIII and MUC4 locus; Figures S1–S12, TALE staining images, quantifications of fluorescence intensities, bisulfite sequencing traces; epitope tag sequences; plasmid maps (PDF)
