# Supplementary material for: Imaging-Based In Situ Analysis of 5-Methylcytosine at Low Repetitive Single Gene Loci with Transcription-Activator-Like Effector Probes
Source: ACS Chem Biol. 2023 Jan 24;18(2):230–6. doi: 10.1021/acschembio.2c00857 (PMC9942090; doi:10.1021/acschembio.2c00857)
Supplement: Supplementary file 1 — cb2c00857_si_001.pdf [file cb2c00857_si_001.pdf]

# Supporting Information

# Imaging-based in situ Analysis of 5-Methylcytosine at Low Repetitive Single Gene Loci with Transcription-Activator-Like Effector Probes

Anne Jung[1], Álvaro Muñoz-López[1,2], Benjamin Buchmuller[1,2], Sudakshina Banerjee[1,2], and Daniel Summerer*[1,2]

1 Faculty of Chemistry and Chemical Biology, TU Dortmund University, Otto-Hahn-Str. 6, 44227 Dortmund (Germany)
2 International Max Planck Research School of Chemical and Molecular Biology,Otto-Hahn-Str. 11, 44227 Dortmund (Germany)

# Table of Contents


Material and Methods ........ 1

- Plasmid cloning ........ 1
- TALE expression and purification ........ 2
- Mammalian cell transfection with DNMT3a3L ........ 2
- Flow cytometry and cell sorting ........ 3
- TALE and antibody staining ........ 3
- Microscopy ........ 4
- Image processing and analysis ........ 4
- Data analysis and statistics ........ 4
- Methylated DNA immunoprecipitation and qPCR ........ 4
- gDNA isolation, bisulfite conversion and Sanger sequencing ........ 5

Oligonucleotide tables ........ 5

- Table S1: Oligonucleotides for cloning ........ 5
- Table S2: PCR primer ........ 6

TALEs assembly ........ 6

- Table S3: TALE RVD composition ........ 6

Statistical data ........ 7

- Table S4: Statistical data of Fluorescence signal amplification experiment (Fig. 2c) .... 7
- Table S5: Theoretical target sequences of TALE_SatIII in each chromosome ........ 8
- Table S6: Theoretical repeats of sequences in MUC4 gene based on GRCh38.p14 and T2T-CHM13v2.0 genome assembly ........ 9

Supplementary Figures ........ 10

- Figure S1. Dividing HCT116 DKO cell shows MUC4 foci in mirrored conformation. 10
- Figure S2. Co-stains of different TALEs targeting the MUC4 locus. ........ 10
- Figure S3. GCN4 – Centriolin co-stain. ........ 11
- Figure S4. Analysis of fluorescence signal amplification with TALE_M3. ........ 12
- Figure S5. Signal noise ratio in red and green fluorescence channel. ........ 12
- Figure S6. Employed antibodies in co-stain experiments do not show unspecific off-target binding. ........ 13
- Figure S7. TALE_M3 co-stains of HCT116 wt and HCT116 DKO cells with generated masks from mCherry images. ........ 14
- Figure S8. Fluorescence signal intensities of foci from cells co-stained with G* and HD TALE fused to mCherry or eGFP. ........ 15

- **Figure S9. Fluorescence signal intensities of nuclei background from cells co-stained with G* and HD TALE fused to mCherry or eGFP.**. ..... 16
- **Figure S10. TALE_M3 1x-mCherry and TALE_M3 1x-eGFP co-stains of HCT116 DKO cells transfected with Dnmt3a3L wt or KO.** ..... 17
- **Figure S11. Nuclei background fluorescence intensities of HCT116 wt and DKO cells stained with G* and HD TALEs not showing MUC4 foci.** ..... 18
- **Figure S12. Sanger sequencing traces of PCR product from bisulfite converted DNA from HCT116 wt and HCT116 DKO cells.** ..... 19

**Supplementary References** ..... 19

**Appendix** ..... 20

- **Epitope Tag Sequences** ..... 20
- **Plasmid Maps** ..... 21

## Material and Methods

### Plasmid cloning

Plasmid pAnJ1861, for expression of TALEs fused to three N-terminal mCherry, was cloned by Gibson assembly[1]. For this, TALE expression vector pAlM1577[2], containing N-terminal mCherry, was linearized using primer o3661 and o3662. The two inserts mCherry_2 and mCherry_3 were amplified using o3657 and o3658 for mCherry_2 and o3659 and o3660 for mCherry_3. Gibson assembly was performed with a 2:1 insert:vector molar ratio.

To generate plasmids for expression of TALEs fused to 20 or 30 N-terminal FLAG tags, restriction sites AgeI and NheI were introduced into vector pAnI521[3] via QuikChange site-directed mutagenesis (Agilent) using primers o4468 and o4469 for AgeI and o4470 and o4471 for NheI. A pre-existing AgeI site was removed using o4472 and o4473 via site-directed mutagenesis. To generate pPiB2670, containing a 10x-FLAG array, two inserts containing 5x-FLAG sequences each (see Appendix for sequences), were amplified using o4493 and o4494 for insert_1 and o4495 and o4496 for insert_2. For following ligation, modified vector pAnI521 was restricted with AgeI and NheI, insert_1 with AgeI and BamHI and insert_2 with BamHI and XbaI. Vector and insets were ligated with T4 ligase (New England Biolabs, #M0202T) using a 3:1 insert:vector molar ratio. Site-directed mutagenesis using o4587 and o4588 was performed to introduce a start codon. For generation of pPiB2683 containing a 20x-FLAG array, modified pPiB2670 was restricted with AgeI and NheI and ligated with restricted insert_1 and 2 as described above. For cloning of pPiB2700 containing a 30x-FLAG array, the before described restriction and ligation was repeated with pPiB2683 as vector.

The plasmid pCrW2056 for expression of TALEs fused to 24 N-terminal GCN4 tags was cloned by amplification of the 24x-GCN4 insert from pAlM1103 (Adgene, #60910) using primers o3935 and o3936. The vector pAnI521 was linearized with restriction enzymes NdeI and NotI and ligated to the insert by Gibson assembly with a 2:1 insert:vector molar ratio.

TALEs were assembled as previously described by Golden Gate Assembly[4] (see Table X for detailed RVD composition). To generate plasmids coding for TALE proteins in frame with a C-terminal His6-Tag and different N-terminal tags, the plasmids pAnI521 for 1x-GFP, pAlM1577 for 1x-mCherry, pAnJ1861 for 3x-mCherry, pPiB2683 for 20x-FLAG, pPiB2700 for 30x-FLAG and pCrW2056 for 24x-GCN4 were used as entry vectors in Golden Gate 2 reactions.

To generate the active DNMT3a3L vector pCoT3181, plasmids pAlH1894 and pJaW876[2] were restricted with AcsI and RcoRI and ligated with T4 ligase using a 2:1 insert:vector molar ratio. Generated plasmid pCoT3180 was linearized with NotI and ligated by Gibson assembly with the CMV-EBFP2 insert, amplified from plasmid EBFP2-N1 (Adgene, #54595) with primers o5028 and o5029, resulting in pCoT3181. Mutation E756A for catalytically inactive DNMT3a3L was introduced by site-directed mutagenesis using primers o2038 and o2039 to generate pAnJ3188.

## TALE expression and purification

TALEs were expressed and purified as described previously[5]. Briefly, TALE plasmids were transformed in electrocompetent BL21 DE3 Gold *E.coli* cells and grown on LB carbenicillin (Carb, 100 mg/mL) agar plates at 37 °C overnight. 5 mL LB medium supplemented with 100 mg/mL carbenicillin were inoculated with a single colony and incubated for 4 h at 37 °C and 220 rpm. This starter culture was transferred to a flask containing 100 mL of LB + Carb and incubated under the same conditions until a $OD_{600}$ of 0.6 arbitrary units (au) was reached. TALE expression was induced by addition 0.4 mM IPTG. For expression of TALEs fused to mCherry tags, cultures were incubated at 18 °C and 220 rpm overnight. Expression cultures of TALEs fused to GFP tag, FLAG tags or SunTag were incubated at 37 °C and 220 rpm for 4 h. Cells were harvested by centrifugation at 3000 g at 4 °C for 20 min. The pellet was kept at -20 °C for 2 h and resuspended in 10 mL Deep Lysis Buffer (10 mM Tris-HCl, 300 mM NaCl, 2.5 mM $MgCl_2$, 5 % DMSO, 0.2 % sodium lauroyl sarcosinate (AppliChem), 0.1 % Triton X-100, pH = 9) containing 1 mM PMSF, 1 mM DTT and 50 µg/mL lysozyme (Sigma Aldrich). Cell lysis was aided by sonication on ice (3 min; 20 % amplitude; 4s on, 2s off). Samples were centrifuged at 14000 g at 4 °C for 20 min to remove cell debris. The supernatant was incubated with 0.5 mL HisPur™ Ni-NTA Resin (ThermoFisher Scientific, #88221) overnight at 4 °C spinning on a rotating wheel. The beads were collected and washed with PBS, twice with Lysis buffer (10 mM Tris-HCl, 300 mM NaCl, 2.5 mM $MgCl_2$, 0.1 % Triton X-100, pH = 9) + 20 mM imidazole + 1mM DTT and three times with Lysis buffer + 50 mM imidazole + 1mM DTT. TALEs were eluted by incubating the beads with 1 mL Lysis buffer + 500 mM imidazole + 1 mM DTT shaking at 800 rpm at 4 °C overnight. Samples were centrifuged at 12000 g for 5 min and the supernatant was purified with Amicon™ Ultra-0.5 Centrifugal Filter units (Merck, MWCO: 100 kDa, #UFC510024) by centrifugation at 14000 g for 10 min at 4 °C. For washing, the volume of the sample was filled up to 500 µL with TALE Storage buffer (200 mM NaCl, 20 mM Tris, 10% glycerol, pH = 7.5) + 1 mM DTT and centrifuged at 14000 g for 10 min at 4 °C. Washing was repeated three times. Samples were recovered by centrifugation at 1000 g for 2 min and filled up with TALE Storage Buffer to a volume of 500 µL. Samples were centrifuged at 14000 g at 4 °C for 5 min and aliquots were snap-frozen with liquid nitrogen and stored at -80 °C. Protein concentrations were measured by BCA using Microplate BCA Protein Assay Kit – Reducing Agent Compatible (ThermoFisher Scientific, #23252) following manufacturer's instructions.

## Mammalian cell transfection with DNMT3a3L

1 million HCT116 DKO cells (DNMT1 (Δexons3-5/Δexons3-5), DNMT3B (-/-), Horizon Discovery Ltd., #HD R02-022) were seeded on 10 cm diameter dishes (Sarstedt, #83.3902.300) in 10 mL full RPMI medium (RPMI 1640 (with L-Glu) (PanBiotech, #P04-16500) + 10% FBS (PanBiotech, #P30-3302) + 1% Pen/Strep (PanBiotech, #P06-07050)) and incubated at 37 °C and 5% $CO_2$ for three days. For transfection, 36 µL FuGENE HD transfection reagent (Promega, #E2311) was mixed with 564 µL OptiMEM (Gibco) and incubated for 5 min at RT. 12 µg (120 µL of 100 ng/µL solution) plasmid pCoT3181 or pAnJ3188 was added, mixed by pipetting up and down and kept for 15 min at RT. The medium of the cells was replaced with 2500 µL fresh prewarmed full RPMI medium and the transfection mixture was added dropwise to the cells. The cells were placed for 1.5 h in the incubator, then 8 mL pre-warmed full RPMI

medium was added. The cells were further incubated for 48 h at 37 °C and 5% $CO_2$ before sorting.

### Flow cytometry and cell sorting

Transfected HCT116 DKO cells were washed with DPBS (PanBiotech, P04-361000), trypsinized with Trypsin 0.05% / EDTA 0.02% (PanBiotech, #P10-038100) for 3 min at 37 ºC and blocked with full RPMI medium. Cells were pelleted by centrifugation at 200 g for 5 min at 37 °C, washed with 5 mL DPBS and resuspended in 1.5 mL prewarmed DPBS + 1% BSA. Cells were sorted with a Sony Cell Sorter model LE-SH800SFP in targeted mode using the 405nm laser (filter FL1 450/50, Optical Filter Pattern 2) to detect EBFP2 transfection control from DNMT3a3L plasmids. Gates were set to assure similar expression levels of fluorescent protein in active and inactive DNMT3a3L transfected samples. EBFP2+ cells were collected in tubes containing prewarmed full RPMI medium. For microscopy, 40.000 cells/well were seeded on μ-Plate 96 Well Black ibiTreat tissue culture treated plates (ibidi, #89626) (coated with 0.01 % poly L-lysine in DPBS for 1 h at 37°C) and incubated for 16 h at 37 °C and 5% $CO_2$.

### TALE and antibody staining

19.000 U2OS (Sigma Aldrich, #92022711-1VL) cells, 32.000 HCT116 wt cells or 32.000 HCT116 DKO cells per well were seeded on μ-Plate 96 Well Black ibiTreat tissue culture treated plates and incubated overnight at 37 °C and 5% $CO_2$ in growth medium (DMEM (w/o L-Glu) (PanBiotech, #P04-03609) + 10% FBS + 1% Pen/Strep + 1% L-Glu (PanBiotech, #P04-80100) for U2OS cells or RPMI 1640 medium (with L-Glu) + 10% FBS + 1% Pen/Strep for HCT116 cells). On the next day cells were washed with DPBS and fixed with ice-cold methanol at -20 °C for 10 min. Cells were washed with DPBS and treated with 2N HCl for 5 min at RT followed by three washing steps with DPBS at 450 rpm for 5 min each. Cells were blocked with Blocking Buffer (DPBS + 1% BSA + 0.1% Tween20) at 450 rpm at RT for 2 h. TALE staining was performed with 200 μL of 1 nM purified TALE in Blocking Buffer for 30 min at 450 rpm at RT. Cells were washed three times with Blocking Buffer for 5 min at RT shaking at 450 rpm and kept in Blocking Buffer at 4 °C, shaking at 450 rpm overnight. For antibody staining, all primary antibodies were diluted 1:1000 in Blocking Buffer, 200 μL was added to each well and incubated for 1 h, shaking at 450 rpm at RT. Cells were washed three times with Blocking Buffer for 5 min, 450 rpm each. All secondary antibodies were diluted 1:2000 in Blocking Buffer and 200 μL were added to each well for 1 h at 450 rpm, RT. Cells were washed three times with Blocking Buffer for 5 min, 450 rpm each. For anti-RFP Booster staining, RFP Booster was diluted 1:200 in Blocking Buffer. Staining and washing was performed as described before. Nucleus staining was performed by incubating the samples with 2 μL per well of Vectashield with DAPI (Vector Laboratories, #H-1200) in 200 μl DPBS for 10 min at 450 rpm, RT. Each well was washed with DPBS for 5 min at 450 rpm, RT and kept in DPBS for microscopy.

**Microscopy**

Experiments were performed using an Olympus IX81 microscope equipped with LEDs as excitation light source (150–750 mW) coupled with a Hamamatsu model C10600-10B-H camera. Images were acquired using a 60x oil objective with compatible immersion oil (ibidi, #50101). Z-stack images (0.3 µm/step, range: 8 µm) for DAPI (excitation filter 395/25 nm, emission filter 474/27 nm), EGFP (excitation filter 475/28 nm, emission filter 554/23 nm) and mCherry (excitation filter 555/28 nm, emission filter 635/18 nm) were taken.

**Image processing and analysis**

Image processing and analysis was performed as described previously[2]. The intensity and subcellular localization of foci was analyzed from z-projections of image stacks (1344 × 1024 pixels, 12 bits) with maximal intensity using the FIJI distribution of ImageJ. To subtract the background, the mean intensity of an out-of-interest region was measured from each channel and subtracted from the stack. Nuclear regions were selected from DAPI images (10 $\mu m^2$ minimum area, circularity between 0.5-1.0). To analyze intensity and size of the foci in mCherry images, the "GaussFit OnSpot" plugin was applied, using elliptical shape and Levenberg Marquard fit mode with a rectangle half size of 10 pixel. Spots larger than 12 pixel or outside the nuclear regions were excluded and the prominence (signal-to-noise ratio) was adjusted for each condition to only select foci-like objects. The generated mask from mCherry images was applied to the eGFP images to measure the mean fluorescence intensity. Image processing was performed in batch, utilizing an ImageJ macro script. For each nucleus, the number, size, and intensity of the associated foci in the mCherry and EGFP images was recorded.

**Data analysis and statistics**

Data analysis and plotting was performed with R as described previously[2]. For each TALE, the log transformed mean fluorescence intensity of each focus was normalized to the average fluorescence intensity of all foci from the associated HCT116 DKO or DNMT3a3L KO transfected sample for each experiment. Graphs were plotted using the ggplot2 library. For statistical analysis a Stutdent's t-test was applied with GraphPad, considering the number of independent experiments as sample size ($N \geq 3$ independent experiments in every case).

**Methylated DNA immunoprecipitation and qPCR**

DNA from HCT116 cells was isolated and purified using the QIAmp DNA Mini Kit (Qiagen, #51304) following the manufacturer's instructions. 300 µL (10 ng/µL) purified DNA was sheared into fragments around 400 bp, using the Bioruptor Pico (Diagenode, #B01080010) in 1.5 mL Bioruptor Microtubes (Diagenode, #C30010016). Samples were sonicated for 11 cycles (30 sec on, 30 sec off) at 4 °C. Fragment size was analyzed with the BioAnalyzer (Agilent) and DNA was concentrated to a concentration of 100 ng/µL using the Concentrator plus (Eppendorf). For methylated DNA immunoprecipitation and qPCR, the MagMeDIP qPCR Kit

(Diagenode, #C02010021) was used according to the manufacturer's instructions. qPCR was performed on the CFX384 Touch real-time PCR detection system (Bio-Rad).

**gDNA isolation, bisulfite conversion and Sanger sequencing**

DNA from HCT116 wt and DKO cells was isolated and purified using the QIAmp DNA Mini Kit (Qiagen, #51304) following the manufacturer's instructions. Bisulfite conversion was conducted with 1 ng DNA per reaction, using the EpiTect Bisulfite Kit (Qiagen #59104) according to the manufacturer's instructions. A 157 bp locus within the MUC4 locus was amplified from bisulfite-converted gDNA using OneTaq DNA polymerase (NEB #M0480S) and primers o5262 and o5263. Amplified DNA was purified with the Monarch PCR & DNA Cleanup Kit (NEB #T1030L) and sequenced using primer o5262 as forward primer and o5262 as reverse primer.

**Oligonucleotide tables**

**Table S1: Oligonucleotides for cloning**

| Name | Sequence (5'→ 3') |
|---|---|
| o2038 | CCCTTCTTCTGGCTCTTTGCCAATGTGGTGGCCATGGGCG |
| o2039 | CCATGGCCACCACATTGGCAAAGAGCCAGAAGAAGGGGCG |
| o3579 | TTTAACTTTAAGAAGGAGATATACAGGATCCGGAAGTATGCGTAAAG |
| o3580 | CGAATAACCGAGTGTCTTATCTAGATTACCTGCCTCGAGTTTGTACAGT |
| o3657 | TCTGGCGGCCGCTCTGGCATGGTGAGCAAGGGCGAGGAGG |
| o3658 | GCTACCATCGATGCTACCCTTGTACAGCTCGTCCATGCCG |
| o3659 | GGTAGCATCGATGGTAGCATGGTGAGCAAGGGCGAGGAGG |
| o3660 | TTGCGAATAACCGAGTGTCTTGTACAGCTCGTCCATGCCG |
| o3661 | ACACTCGGTTATTCGCAA |
| o3662 | GCCAGAGCGGCCGCCAGACTTGTACAGCTCGTCCATGCCG |
| o3935 | TTTAACTTTAAGAAGGAGATATACAGGATCCAACGGTCCGGG |
| o3936 | CGAATAACCGAGTGTCTTACCCGAGCCAGAACCCTTT |
| o4468 | GAAGGAGATATACCGGTGAGCAAGGGCGAGGAGCTGTTCACCGGG |
| o4469 | CCCGGTGAACAGCTCCTCGCCCTTGCTCACCGGTATATCTCCTTC |
| o4470 | CTCTCGGCATGGACGAGCTAGCCAAGACACTCGGTTATTCGCAAC |
| o4471 | GTTGCGAATAACCGAGTGTCTTGGCTAGCTCGTCCATGCCGAGAG |
| o4472 | GCTCACTGCCCGCTTTCCACTGGTCGTCTCCAACGACCATCTGCT |
| o4473 | AGCAGATGGTCGTTGGAGACGACCAGTGGAAAGCGGGCAGTGAGC |
| o4493 | ATCCCGACCGGTAAGCTGCT |
| o4494 | TAATCGGATCCGCCTTTGTC |
| o4495 | AAGGCGGATCCGATTACAAA |
| o4496 | CGGCTCTAGACCTTTGTCAT |
| o4587 | GTTTAACTTTAAGAAGGAGATATACCATGAAGCTGCTAGCCTCGG |
| o4588 | CCGAGGCTAGCAGCTTCATGGTATATCTCCTTCTTAAAGTTAAAC |
| o5028 | CGACGGATCGGGAGCCGTTACATAACTTACGGTAAATGGCCC |
| o5029 | GATGTCTGCTCGAAGCGCGGCCGCTTACT |

## Table S2: PCR primer

### qPCR

| Locus | Name | Sequence (5'→ 3') |
|---|---|---|
| SatIII | o2419_fw | AATCAACCCGAGTGCAATCGAATGGAATCG |
| | o2420_rv | TCCATTCCATTCCTGTACTCGG |
| MUC4 | o5181_fw | CCTCTTCATGTCACCGATGCTTCCTC |
| | o5182_rv | GGTGTGACCTGTGGATACTGAGGAAGG |

### Bisulfite PCR

| Locus | Name | Sequence (5'→ 3') |
|---|---|---|
| MUC4 | o5262_fw | ATAGGTTATATTATTTTTTTTATGTTATCGA |
| | o5263_rv | TAATATAACCTATAAATACTAAAAAAAAACTAAT |

### TALEs assembly

### Table S3: TALE RVD composition

### TALE_M1

| 1 | 2 | 3 | 4 | 5 | 6 | 7 | 8 | 9 | 10 | 11 | 12 | 13 | 14 | 15 |
|---|---|---|---|---|---|---|---|---|---|---|---|---|---|---|
| NG | NG | HD | HD | NG | HD | NI | NN | HD | NI | NG | HD | HD | NI | HD |

### TALE_M2

| 1 | 2 | 3 | 4 | 5 | 6 | 7 | 8 | 9 | 10 | 11 | 12 | 13 | 14 | 15 | 16 | 17 | 18 |
|---|---|---|---|---|---|---|---|---|---|---|---|---|---|---|---|---|---|
| NG | NN | NN | NG | NN | NI | HD | NI | NN | NN | NI | NI | NN | NI | NN | NN | NN | NN |

### TALE_M3

| | 1 | 2 | 3 | 4 | 5 | 6 | 7 | 8 | 9 | 10 | 11 | 12 | 13 | 14 | 15 | 16 | 17 | 18 |
|---|---|---|---|---|---|---|---|---|---|---|---|---|---|---|---|---|---|---|
| HD | NG | NN | NN | HD | NN | NG | NN | NI | HD | HD | NG | NN | NG | NN | NN | NI | NG | NI |
| G* | NG | NN | NN | G* | NN | NG | NN | NI | HD | HD | NG | NN | NG | NN | NN | NI | NG | NI |

### TALE_SatIII

| | 1 | 2 | 3 | 4 | 5 | 6 | 7 | 8 | 9 | 10 | 11 | 12 | 13 | 14 | 15 | 16 | 17 | 18 |
|---|---|---|---|---|---|---|---|---|---|---|---|---|---|---|---|---|---|---|
| HD | NG | NN | NN | NI | NI | HD | NN | NN | NI | NI | NG | NN | NN | NI | NI | NG | NN | NN |
| G* | NG | NN | NN | NI | NI | G* | NN | NN | NI | NI | NG | NN | NN | NI | NI | NG | NN | NN |

## Statistical data

**Table S4: Statistical data of Fluorescence signal amplification experiment (Fig. 2c)**
Student's *t*-test: $P < 0.1$*, $P < 0.01$**, $P < 0.001$***

| | 3x-mCh | 1x-mCh + Booster | 3x-mCh + Booster | 1x-mCh + 2AB | 3x-mCh + 2AB | 1x-mCh + 3AB | 3x-mCh + 3AB | 20x-FLAG + 2AB | 30x-FLAG + 2AB | 20x-FLAG + 3AB | 30x-FLAG + 3AB | SunTag + 2AB | SunTag + 3AB |
|---|---|---|---|---|---|---|---|---|---|---|---|---|---|
| 1x-mCh | ns | ns | ns | *** | ** | ** | ** | * | * | ** | * | * | *** |
| 3x-mCh | | ns | ns | ** | ** | ** | ** | * | * | * | * | * | ** |
| 1x-mCh + Booster | | | ns | *** | ** | ** | ** | * | * | ** | * | * | ** |
| 3x-mCh + Booster | | | | * | * | * | ** | ns | ns | * | * | * | ** |
| 1x-mCh + 2AB | | | | | ns | * | * | ns | ns | ns | ns | ns | * |
| 3x-mCh + 2AB | | | | | | ns | ns | ns | ns | ns | ns | ns | ns |
| 1x-mCh + 3AB | | | | | | | ns | ns | ns | ns | ns | ns | ns |
| 3x-mCh + 3AB | | | | | | | | * | * | ns | ns | * | ns |
| 20x-FLAG + 2AB | | | | | | | | | ns | ns | ns | ns | * |
| 30x-FLAG + 2AB | | | | | | | | | | ns | ns | ns | * |
| 20x-FLAG + 3AB | | | | | | | | | | | ns | ns | ns |
| 30x-FLAG + 3AB | | | | | | | | | | | | ns | ns |
| SunTag + 2AB | | | | | | | | | | | | | ns |

**Table S5: Theoretical target sequences of TALE_SatIII in each chromosome**

| Chromosome | Target sequences TALE_SatIII |
|---|---|
| 1 | 213 |
| 2 | 29 |
| 3 | 30 |
| 4 | 92 |
| 5 | 289 |
| 6 | 0 |
| 7 | 175 |
| 8 | 0 |
| 9 | 27997 |
| 10 | 231 |
| 11 | 0 |
| 12 | 2 |
| 13 | 625 |
| 14 | 1127 |
| 15 | 4297 |
| 16 | 3 |
| 17 | 443 |
| 18 | 0 |
| 19 | 0 |
| 20 | 395 |
| 21 | 1022 |
| 22 | 1197 |
| x | 0 |
| y | 451 |

**Table S6: Theoretical repeats of sequences in MUC4 gene based on GRCh38.p14 and T2T-CHM13v2.0 genome assembly**

MUC4 TALE target sequences used in this work

| Target sequence | GRCh38.p14 | T2T-CHM13v2.0 |
|---|---|---|
| TGGCGTGACCTGTGGATA | 16 | 32 |
| TGGTGACAGGAAGAGGGG | 46 | 79 |
| TTCCTCAGCATCCAC | 95 | 148 |
| TATCCACAGGTCACGCCA | 16 | 32 |
| TCACGCCACCCCTCTTCC | 25 | 49 |
| TCAGTATCCACAGGTCAC | 18 | 36 |
| TGACCTGTGGATACTGAG | 18 | 38 |
| TTCCTCAGTATCCACAGG | 43 | 76 |
| TGTGGATACTGAGGAAGC | 9 | 20 |

MUC4 TALE or CRISPR-dCas9 target sequences used by others

| Target sequence | GRCh38.p14 | T2T-CHM13v2.0 |
|---|---|---|
| CCTGTCACCGACACTTCC[6,7] | 43 | 67 |
| GACCTGTGGATGCTGAGGAA[8] | 55 | 81 |
| CTTCCTGTCACCGACACTTC[9] | 42 | 65 |
| CAGCATCCACAGGTCACGCCAC[9] | 32 | 44 |
| GTCACCGACACTTCCTCAGCATCCAC[10] | 28 | 43 |
| TCTTCCTGTCACCGACACTTC[10] | 39 | 62 |
| CAGCATCCACAGGTCACGCCAC[10] | 32 | 44 |

## Supplementary Figures

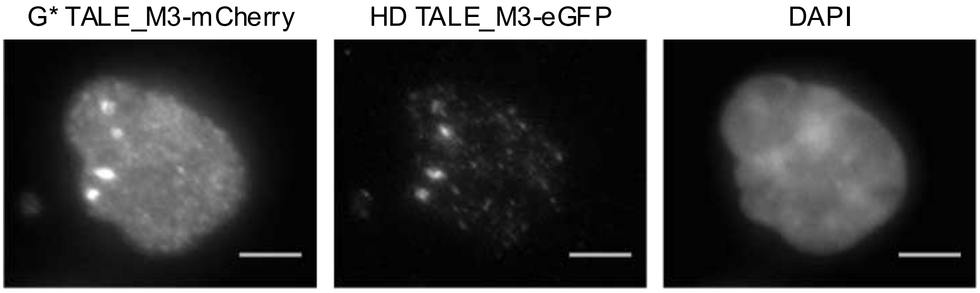


**Figure S1. Dividing HCT116 DKO cell shows MUC4 foci in mirrored conformation.** Cells were co-stained with G* TALE_M3-mCherry and HD TALE_M3-eGFP. Immunostaining with Ms anti-eGFP, Goat anti-Mouse Alexa Fluor 488 and Rb anti-mCherry, Goat anti-Rabbit Alexa Fluor Plus 594. Scale bars: 5 µm.

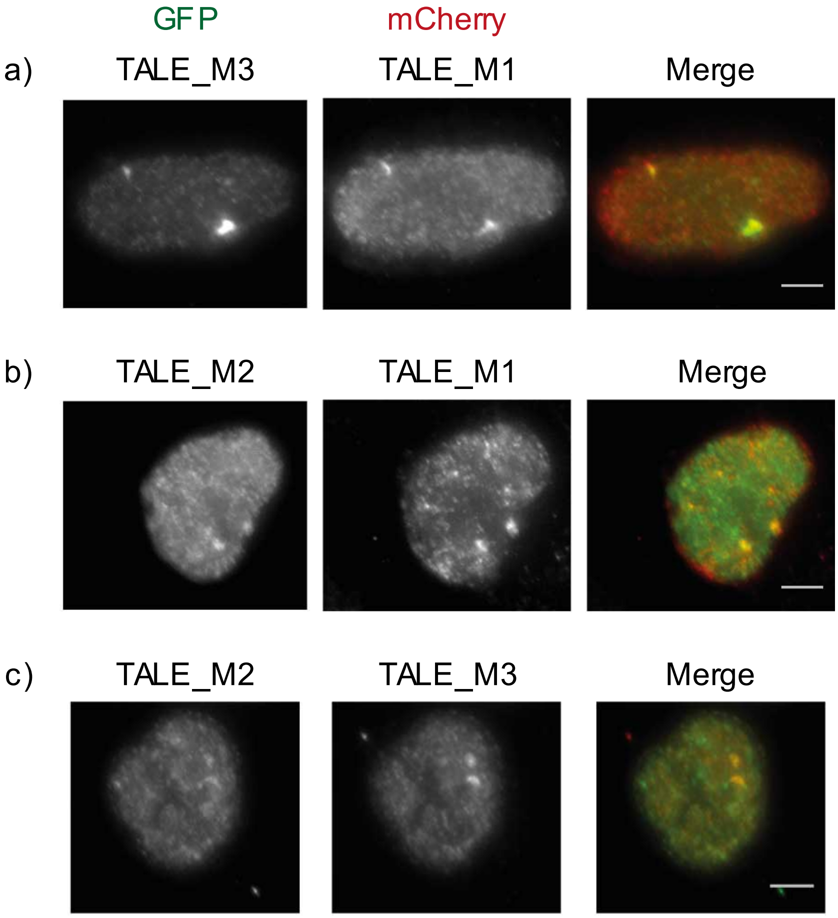


**Figure S2. Co-stains of different TALEs targeting the MUC4 locus.** Co-stains of a) TALE_M3-1x-eGFP and TALE_M1-3x-mCherry b) TALE_M2-1x-eGFP and TALE_M1-3x-mCherry c) TALE_M2-1x-eGFP and TALE_M3-1x-mCherry. Immunostaining with Ms anti-eGFP, Goat anti-Mouse Alexa Fluor 488 and Rb anti-mCherry, Goat anti-Rabbit Alexa Fluor Plus 594. U2OS cells, Scale bars: 5 µm.

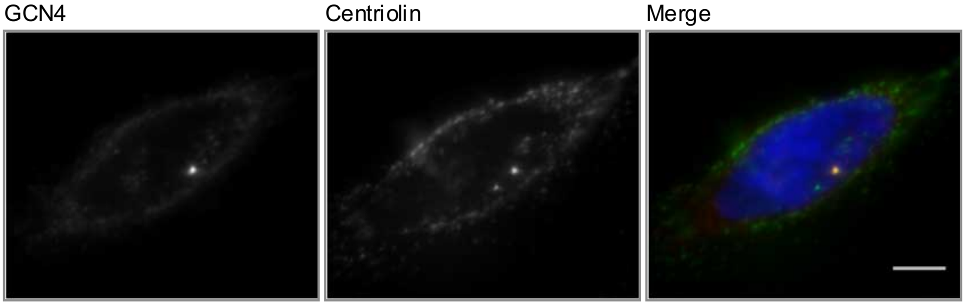


**Figure S3. GCN4 – Centriolin co-stain.** U2OS cell stained with Rb anti-GCN4 (1:500), anti-Rabbit Alexa Fluor plus 594 (1:1000) and Ms anti-Centriolin (1:50), anti-Mouse Alexa Fluor 488 (1:1000). Scale bar: 5 µm.

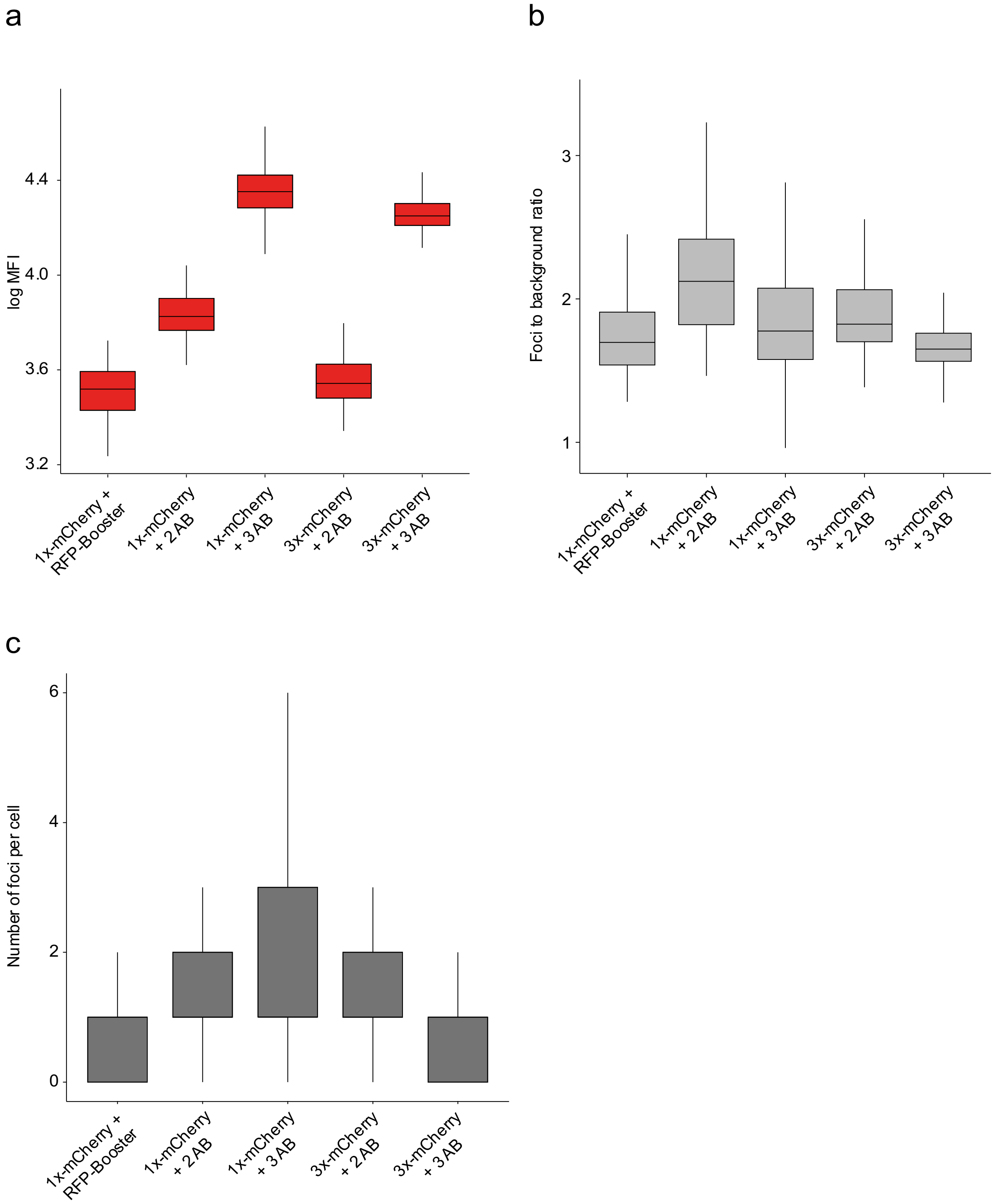

**Figure S4. Analysis of fluorescence signal amplification with TALE_M3.** Fluorescence intensities of foci and nuclear background from experiment shown in Fig 3c. a) log MFI of foci from TALE_M3 staining with different epitope tag and immunostaining combinations. b) MFI ratio of foci to nuclear background. c) Average number of foci per cell.

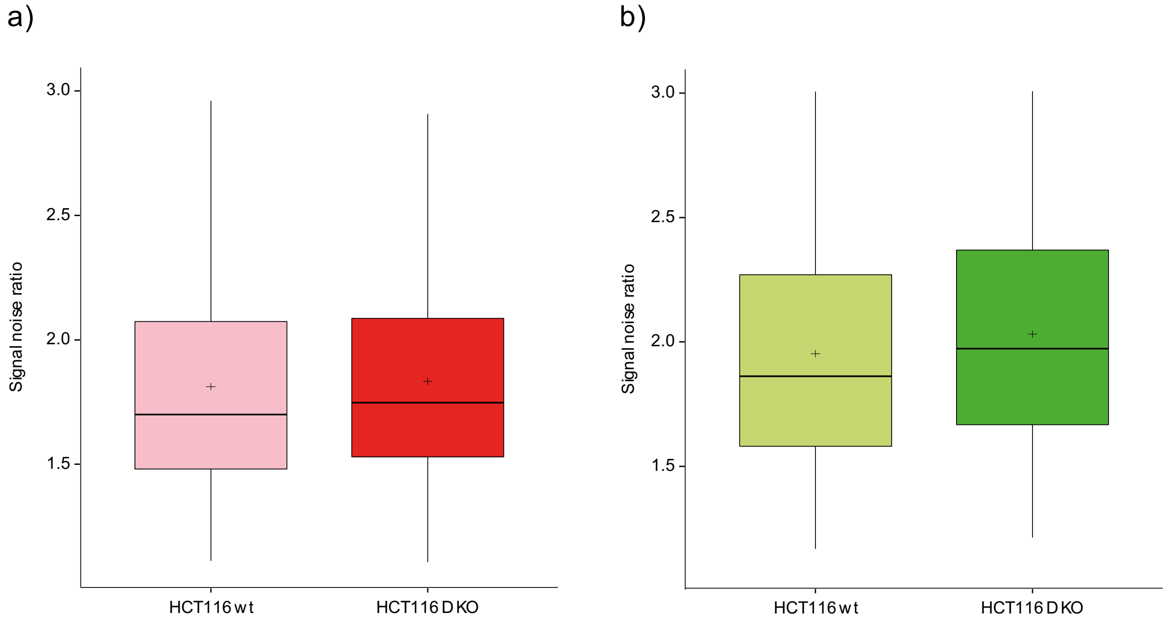


**Figure S5. Signal noise ratio in red and green fluorescence channel.** HCT 116 wt and DKO cells were co-stained with G* TALE_M3-mCherry and G* TALE_M3-GFP and immunostained with Ms anti-eGFP, Goat anti-Mouse Alexa Fluor 488 and Rb anti-mCherry, Goat anti-Rabbit Alexa Fluor Plus 594. Ratio of mean fluorescence intensity of all foci to mean fluorescence intensity of nuclei background is shown for the a) red and b) green channel.

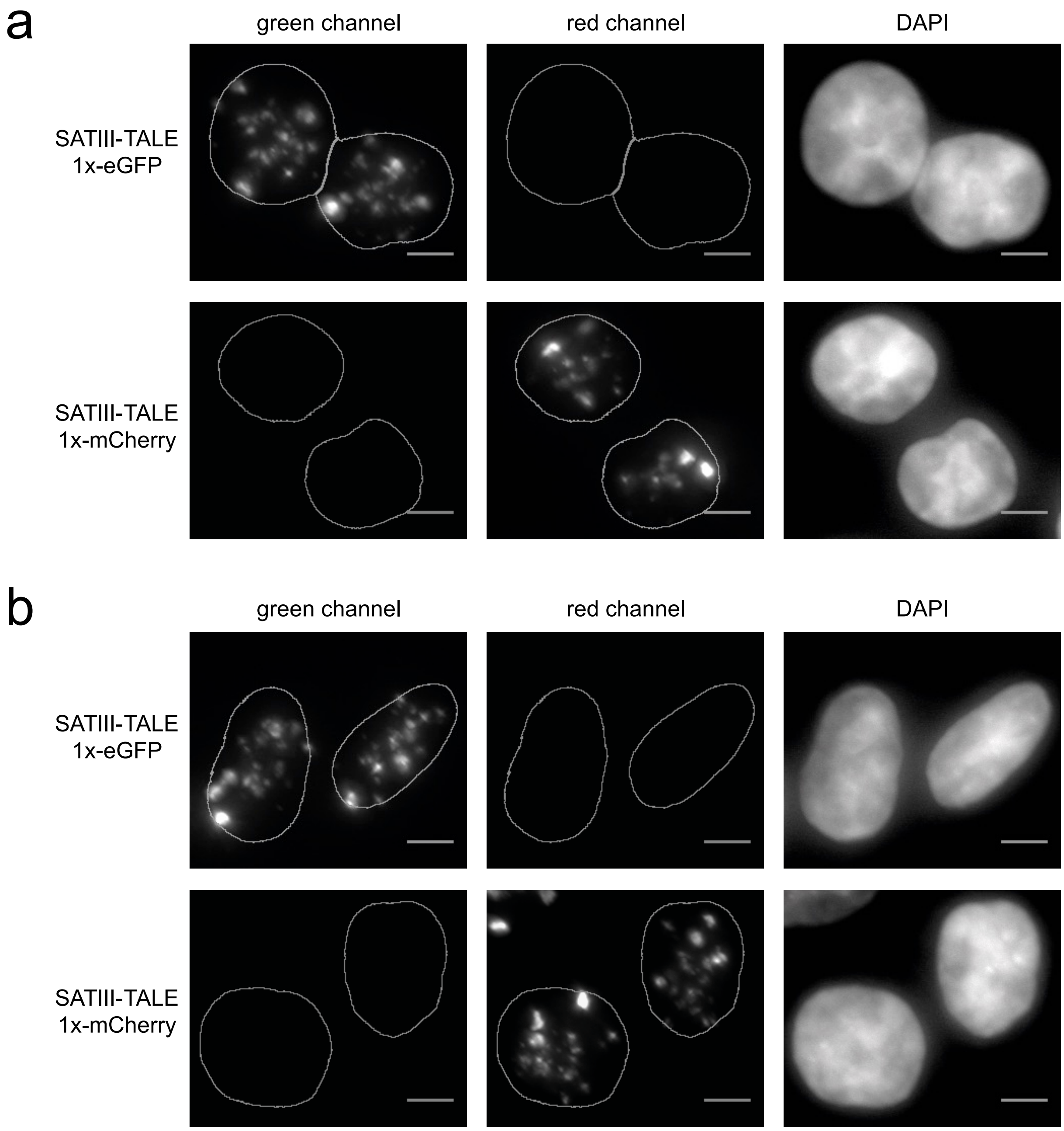


**Figure S6. Employed antibodies in co-stain experiments do not show unspecific off-target binding.** HCT116 wt (a) and HCT116 DKO (b) cells stained with TALE_SatIII 1x-eGFP or TALE_SatIII 1x-mCherry. Cells were immunostained with Ms anti-eGFP, Goat anti-Mouse Alexa Fluor 488 and Rb anti-mCherry, Goat anti-Rabbit Alexa Fluor Plus 594. Fluorescence images of were acquired under the same imaging conditions for each channel. Scale bars: 5 µm.

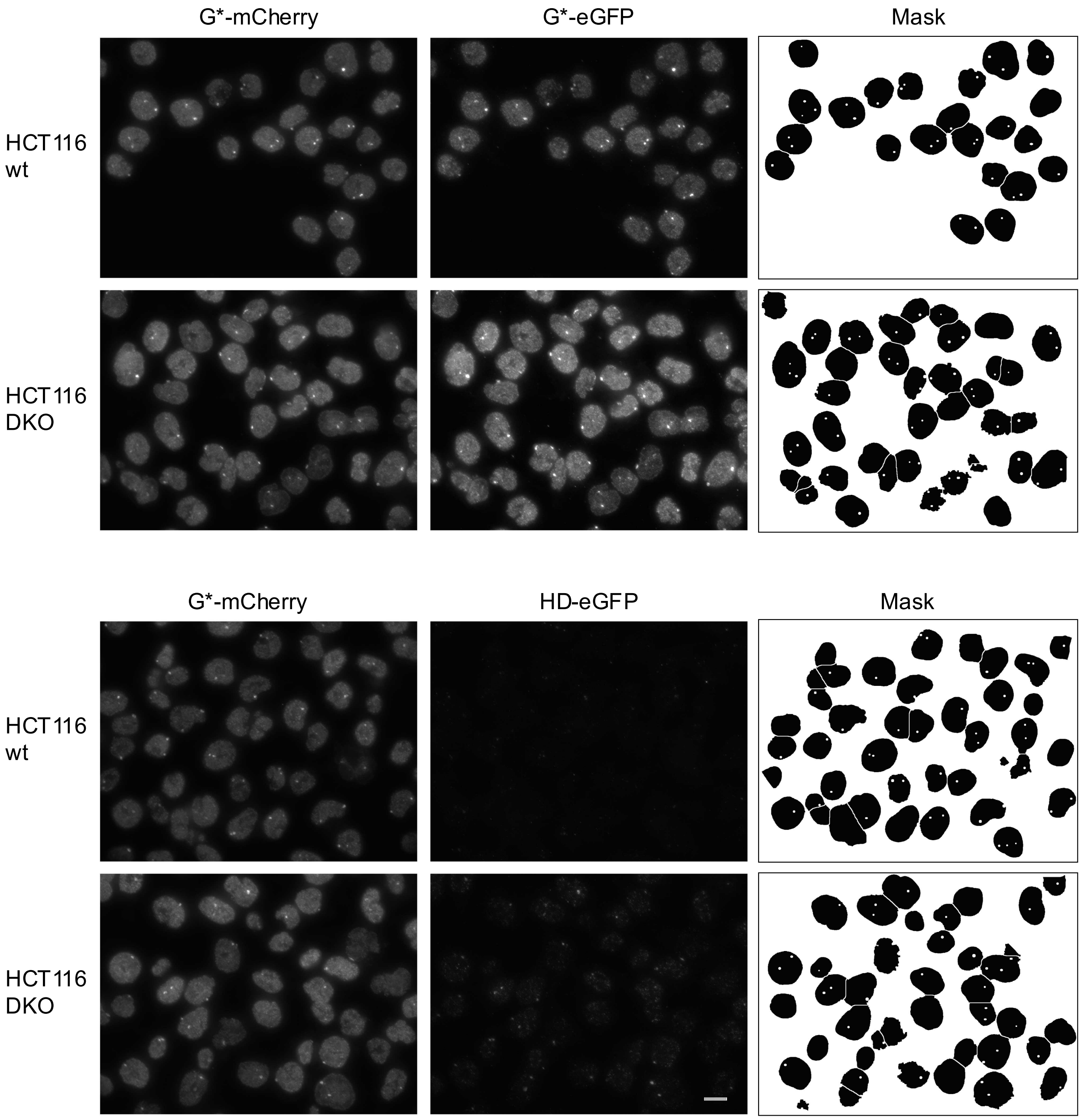


**Figure S7. TALE_M3 co-stains of HCT116 wt and HCT116 DKO cells with generated masks from mCherry images.** Co-stain of G*-mCherry and G*-eGFP TALE on top. Co-stain of G*-mCherry and HD-eGFP TALE below. Fluorescence images of were acquired under the same imaging conditions for each channel. Scale bar: 15 µm.

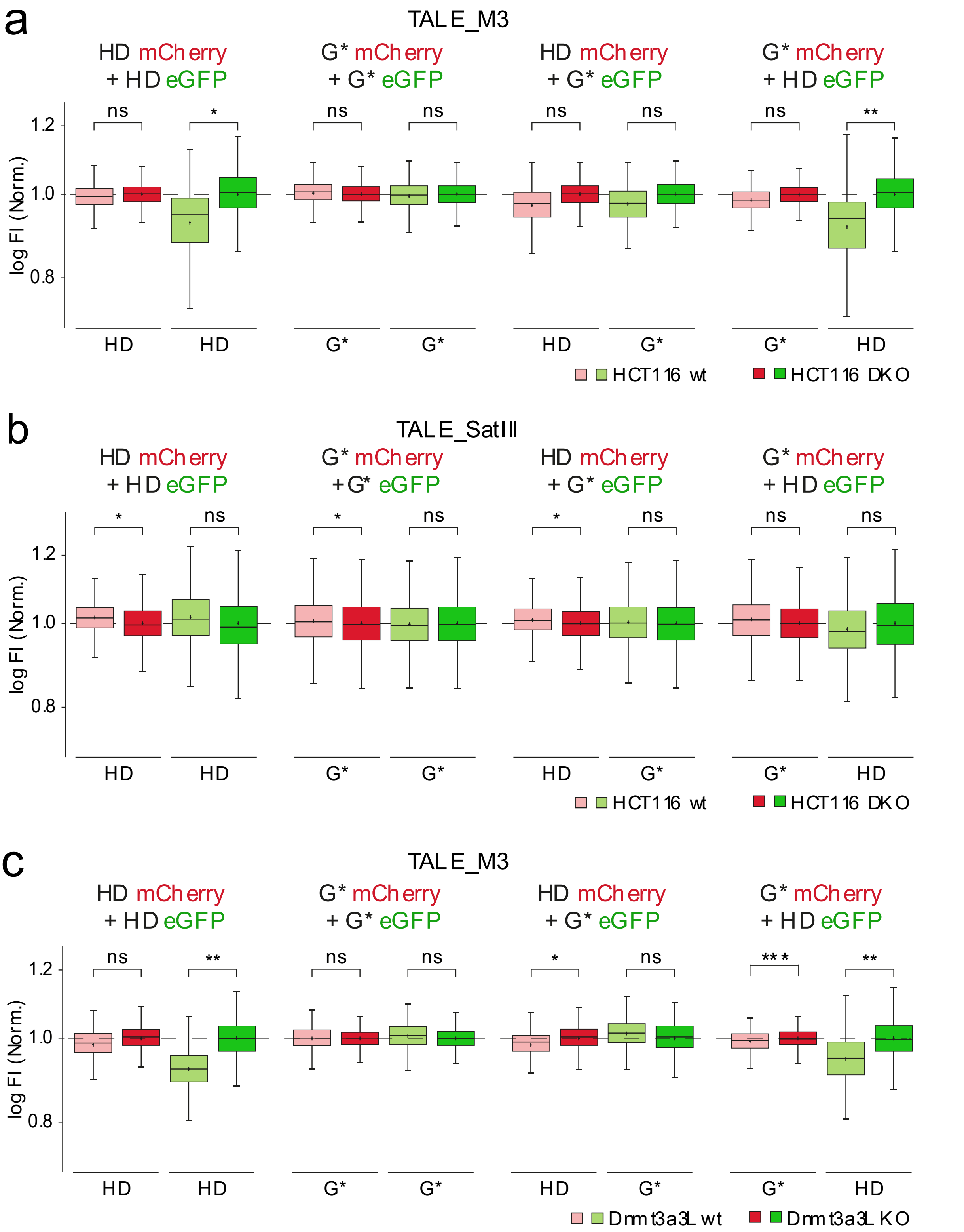


**Figure S8. Fluorescence signal intensities of foci from cells co-stained with G* and HD TALE fused to mCherry or eGFP.** a) TALE_M3 co-stain of HCT116 wt and DKO cells. N = 4 experiments totaling > 2000 foci per condition b) TALE_SatIII co-stain of HCT116 wt and DKO cells. N = 4 experiments totaling > 7000 foci per condition c) TALE_M3 co-stain of HCT116 DKO cells transfected with Dnmt3a3L wt or KO. N = 3 experiments totaling > 1300 foci per condition. For each TALE, log FI of each focus is normalized to the mean of log FI of all foci from HCT116 DKO cells (a, b) or HCT116 DKO cells transfected with Dnmt3a3L KO (c). $P < 0.1$*, $P < 0.01$**, $P < 0.001$*** (Student's *t*-test).

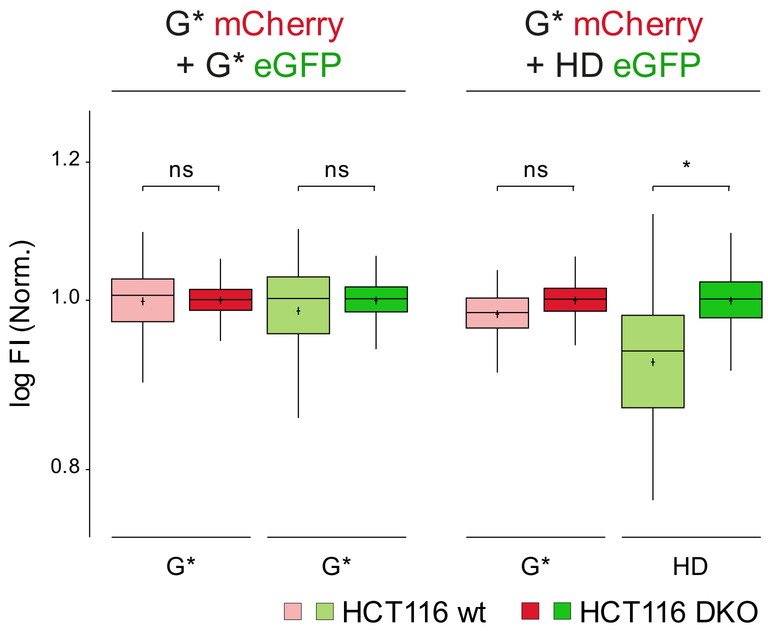


**Figure S9. Fluorescence signal intensities of nuclei background from cells co-stained with G* and HD TALE fused to mCherry or eGFP.** TALE_M3 co-stain of HCT116 wt and DKO cells. N = 4 experiments totaling > 2000 nuclei per condition. For each TALE, log FI of each nucleus is normalized to the mean of log FI of all nuclei from HCT116 DKO cells. $P < 0.1$* (Student's *t*-test).

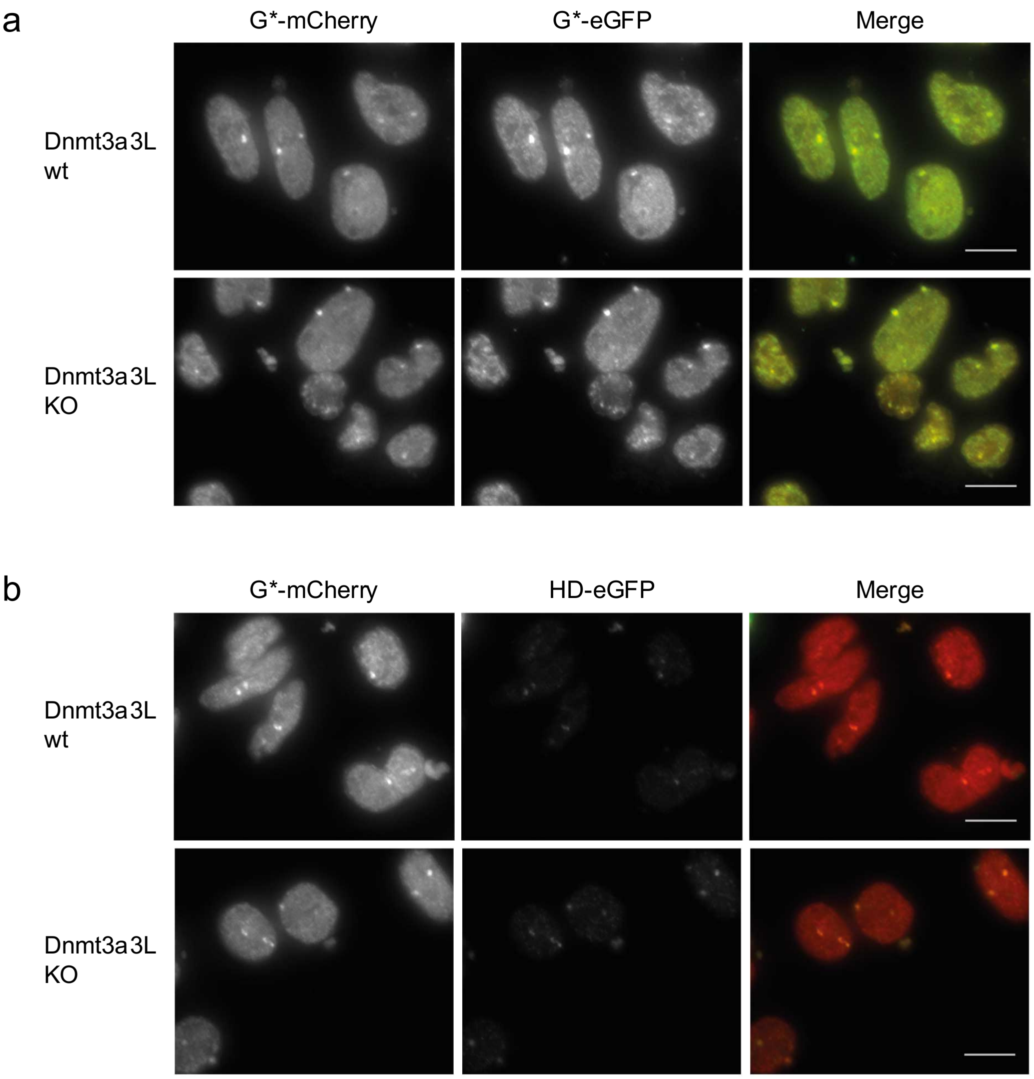


**Figure S10. TALE_M3 1x-mCherry and TALE_M3 1x-eGFP co-stains of HCT116 DKO cells transfected with Dnmt3a3L wt or KO.** a) Co-stain with G*-mCherry and G* eGFP TALE. b) Co-stain with G*-mCherry and HD eGFP TALE. Cells were stained with primary anti-mCherry and anti-eGFP antibodies and secondary Alexa Fluor plus 594 labeled and Alexa Fluor 488 labeled antibodies. Fluorescence images of were acquired under the same imaging conditions for each channel. Scale bars: 10 µm.

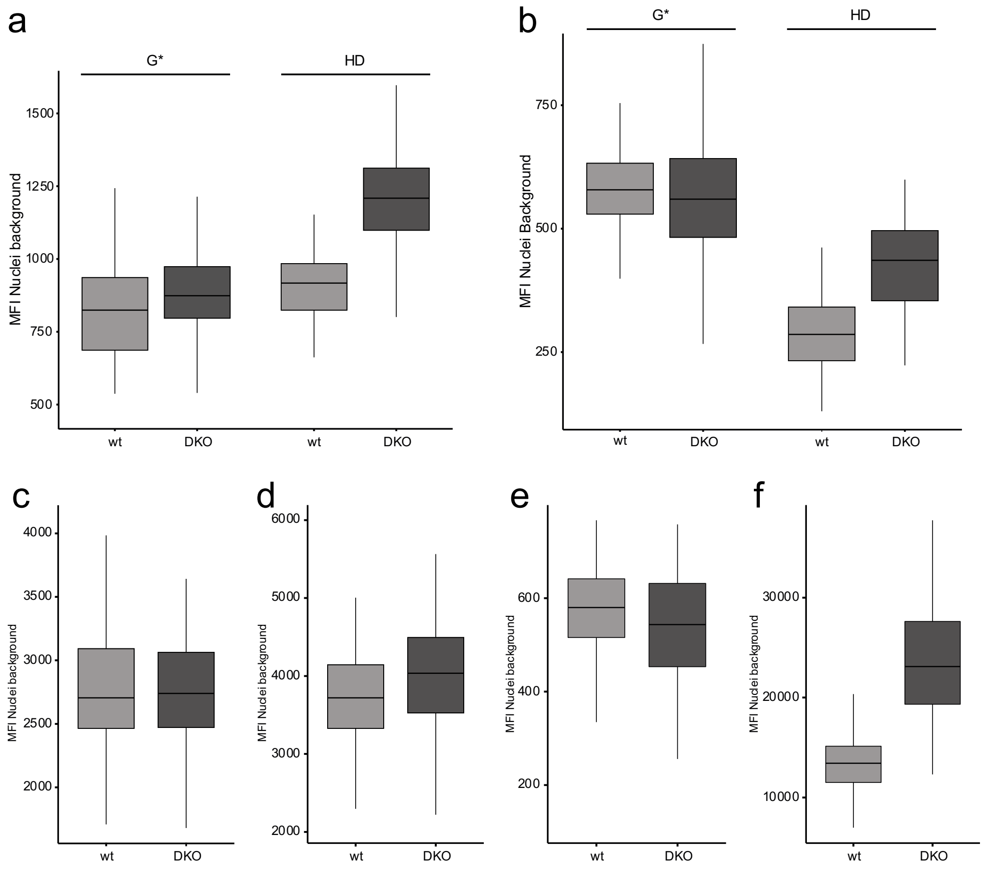


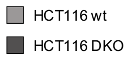


## Target sequences

a) TALE_B1: TATCCACAGGTCACGCCA
b) TALE_B2: TCACGCCACCCCTCTTCC
c) TALE_B3: TCAGTATCCACAGGTCAC
d) TALE_B4: TGACCTGTGGATACTGAG
e) TALE_B5: TTCCTCAGTATCCACAGG
f) TALE_B6: TGTGGATACTGAGGAAGC

**Figure S11. Nuclei background fluorescence intensities of HCT116 wt and DKO cells stained with G* and HD TALEs not showing MUC4 foci.** Staining with single mCherry TALEs with identical size and overall design, but with different, CpG-containing or CpG-free MUC4 target sequences of identical lengths (see table). These TALEs turned out to be weak binders not usable for MUC4 staining with sufficient signal/noise ratios. TALEs differ in absolute nuclear background intensity, suggesting different numbers of off-target sequences. In case of CpG-containing target sequences, only the HD versions showed increased background intensities for the DKO cells, which can be explained by response to mC in off-target sequences containing a CpG opposite the HD position. For CpG-free targets, several TALEs did not show increased background signals in DKO cells, possibly due to an absence of mC in the off-target sequences. TALE single-stainings were followed by immunostaining with Rabbit anti-mCherry and Goat anti-Rabbit Alexa Fluor Plus 594 prior data acquisition.

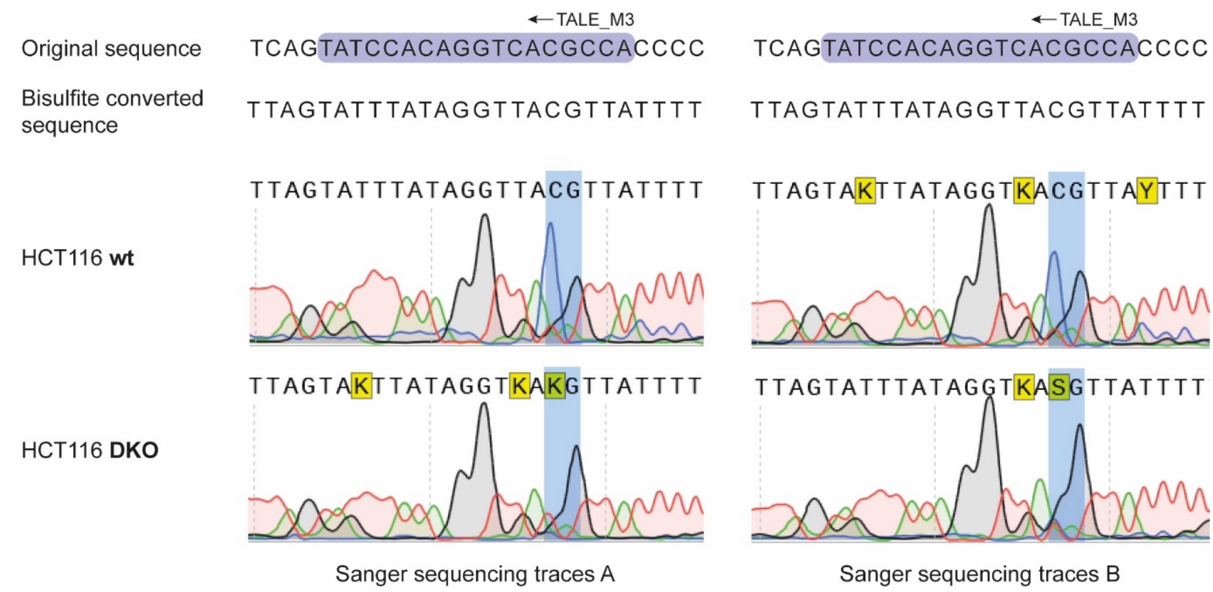


**Figure S12. Sanger sequencing traces of PCR product from bisulfite converted DNA from HCT116 wt and HCT116 DKO cells.** Sanger sequencing traces A and B were obtained from two different PCR products. TALE_M3 target sequence highlighted in purple. CpG position highlighted in light blue.

## Supplementary References

(1) Gibson, D. G.; Young, L.; Chuang, R. Y.; Venter, J. C.; Hutchison, C. A.; Smith, H. O. Enzymatic Assembly of DNA Molecules up to Several Hundred Kilobases. *Nat Methods* **2009**, *6* (5), 343–345. https://doi.org/10.1038/NMETH.1318.

(2) Muñoz-López, Á.; Buchmuller, B.; Wolffgramm, J.; Jung, A.; Hussong, M.; Kanne, J.; Schweiger, M. R.; Summerer, D. Designer Receptors for Nucleotide-Resolution Analysis of Genomic 5-Methylcytosine by Cellular Imaging. *Angew Chem Int Ed Engl* **2020**, *59* (23), 8927–8931. https://doi.org/10.1002/ANIE.202001935.

(3) Gieß, M.; Muñoz-López, Á.; Buchmuller, B.; Kubik, G.; Summerer, D. Programmable Protein-DNA Cross-Linking for the Direct Capture and Quantification of 5-Formylcytosine. *J Am Chem Soc* **2019**, *141*, 8. https://doi.org/10.1021/JACS.9B01432/ASSET/IMAGES/LARGE/JA-2019-01432V_0004.JPEG.

(4) Cermak, T.; Doyle, E. L.; Christian, M.; Wang, L.; Zhang, Y.; Schmidt, C.; Baller, J. A.; Somia, N. v.; Bogdanove, A. J.; Voytas, D. F. Efficient Design and Assembly of Custom TALEN and Other TAL Effector-Based Constructs for DNA Targeting. *Nucleic Acids Res* **2011**, *39* (12), e82. https://doi.org/10.1093/NAR/GKR218.

(5) Kubik, G.; Summerer, D. Achieving Single-Nucleotide Resolution of 5-Methylcytosine Detection with TALEs. *Chembiochem* **2015**, *16* (2), 228–231. https://doi.org/10.1002/CBIC.201402408.

(6) Ren, R.; Deng, L.; Xue, Y.; Suzuki, K.; Zhang, W.; Yu, Y.; Wu, J.; Sun, L.; Gong, X.; Luan, H.; Yang, F.; Ju, Z.; Ren, X.; Wang, S.; Tang, H.; Geng, L.; Zhang, W.; Li, J.; Qiao, J.; Xu, T.; Qu, J.; Liu, G. H. Visualization of Aging-Associated Chromatin Alterations with an Engineered TALE System. *Cell Research 2017 27:4* **2017**, *27* (4), 483–504. https://doi.org/10.1038/cr.2017.18.

(7) Athmane, N.; Williamson, I.; Boyle, S.; Biddie, S. C.; Bickmore, W. A. MUC4 Is Not Expressed in Cell Lines Used for Live Cell Imaging. *Wellcome Open Research 2021 6:265* **2021**, *6*, 265. https://doi.org/10.12688/wellcomeopenres.17229.2.

(8) Qin, P.; Parlak, M.; Kuscu, C.; Bandaria, J.; Mir, M.; Szlachta, K.; Singh, R.; Darzacq, X.; Yildiz, A.; Adli, M. Live Cell Imaging of Low- and Non-Repetitive Chromosome Loci Using CRISPR-Cas9. *Nature Communications 2017 8:1* **2017**, *8* (1), 1–10. https://doi.org/10.1038/ncomms14725.

(9) Chen, B.; Hu, J.; Almeida, R.; Liu, H.; Balakrishnan, S.; Covill-Cooke, C.; Lim, W. A.; Huang, B. Expanding the CRISPR Imaging Toolset with Staphylococcus Aureus Cas9 for Simultaneous Imaging of Multiple Genomic Loci. *Nucleic Acids Res* **2016**, *44* (8), e75. https://doi.org/10.1093/NAR/GKV1533.

(10) Chen, B.; Gilbert, L. A.; Cimini, B. A.; Schnitzbauer, J.; Zhang, W.; Li, G. W.; Park, J.; Blackburn, E. H.; Weissman, J. S.; Qi, L. S.; Huang, B. Dynamic Imaging of Genomic Loci in Living Human Cells by an Optimized CRISPR/Cas System. *Cell* **2013**, *155* (7), 1479–1491. https://doi.org/10.1016/j.cell.2013.12.001.

## Appendix

## Epitope Tag Sequences

**5x-FLAG Sequences**

**Insert_1**

5'ATCCCGACCGGTAAGCTGCTAGCCTCGGACTACAAAGACGACGACGACAAGGG TGGTTCTGATTATAAGGACGATGACGATAAAGGAGGTTCCGACTATAAAGATGAT GATGACAAAGGAGGGTCAGATTACAAGGATGACGACGATAAGGGGGGCTCGGAT TACAAGGACGACGATGACAAAGGCGGATCCGATTA-3'

**Insert_2**

5'AAGGCGGATCCGATTACAAAGACGATGACGACAAGGGTGGATCAGATTATAAG GACGATGATGATAAAGGCGGGTCTGACTATAAAGATGATGATGACAAAGGGGGC TCGGACTACAAGGATGACGACGATAAGGGCGGCTCAGACTATAAGGACGACGAT GACAAAGGTCTAGAGCCG-3'

FLAG sequences are highlighted in gray.

**Plasmid Maps**

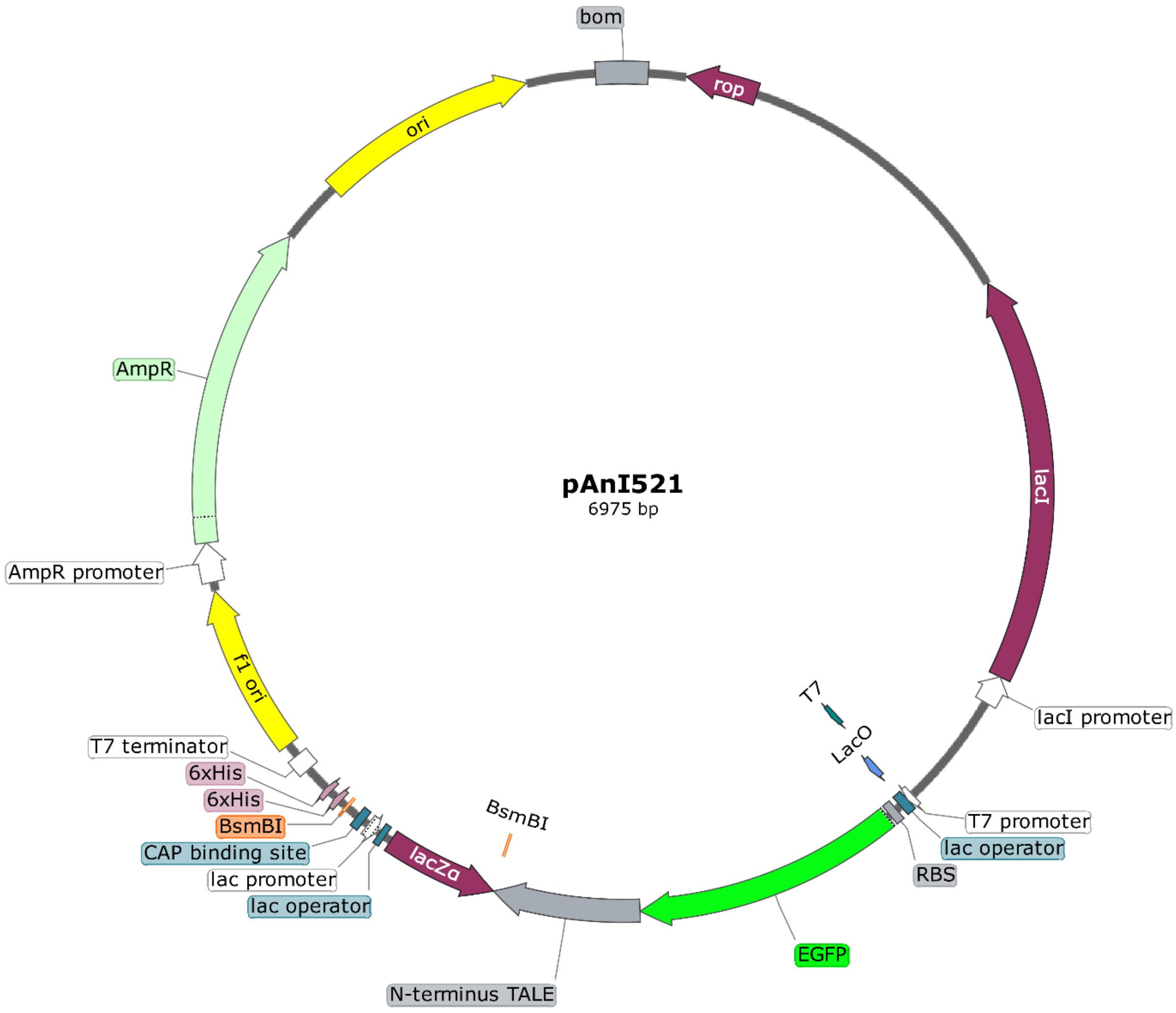


**pAnI521**

GG2 Entry Vector for bacterial expression of TALEs fused to N-terminal EGFP and C-terminal 6xHis for protein purification.

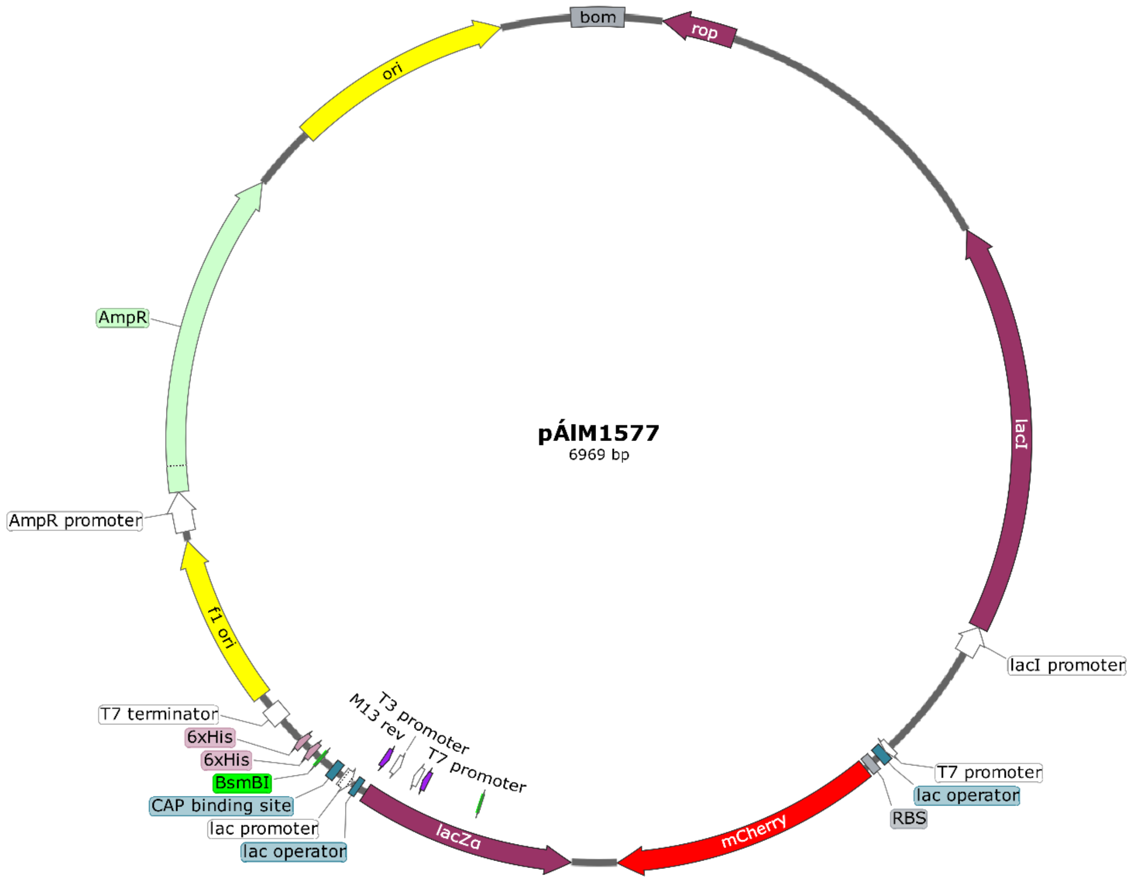


## pAIM1577

Vector for bacterial expression of TALEs fused to N-terminal mCherry and C-terminal 6xHis for protein purification.

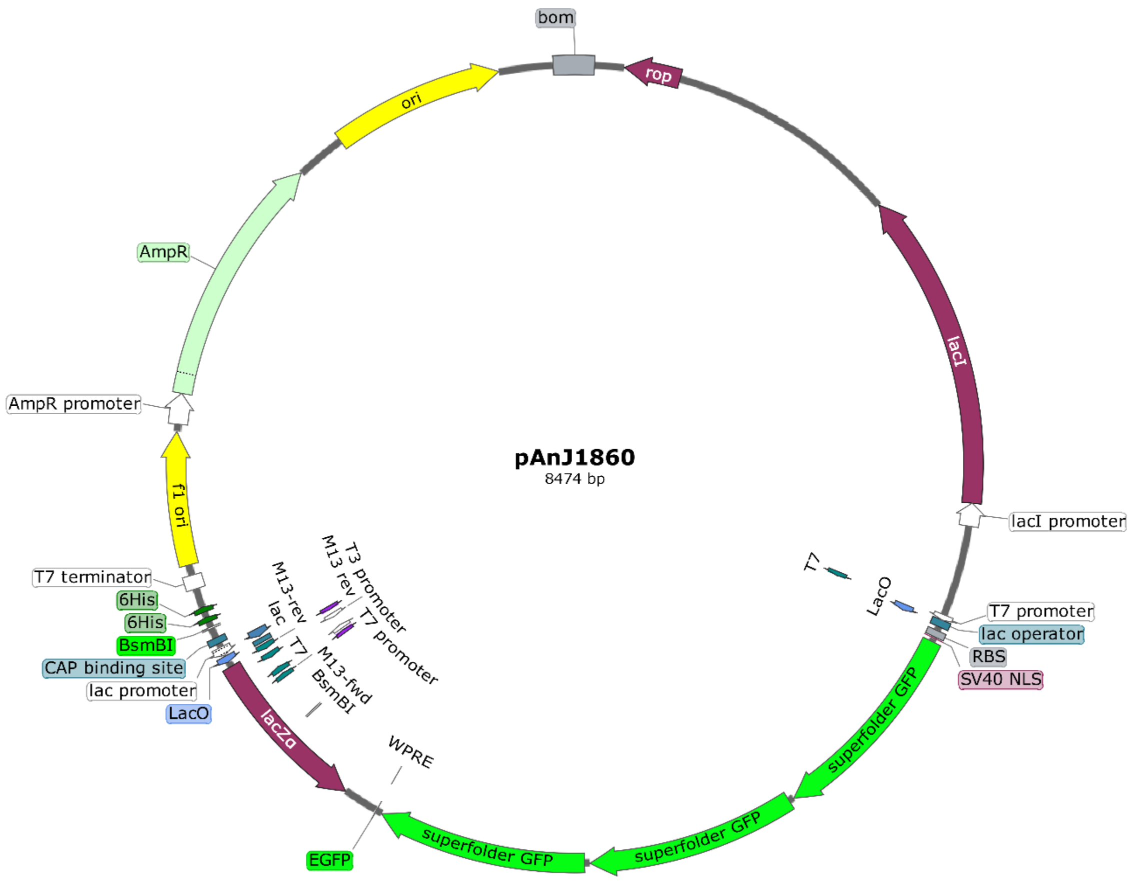


**pAnJ1860**

Vector for bacterial expression of TALEs fused to three N-terminal sfGFP and C-terminal 6xHis for protein purification.

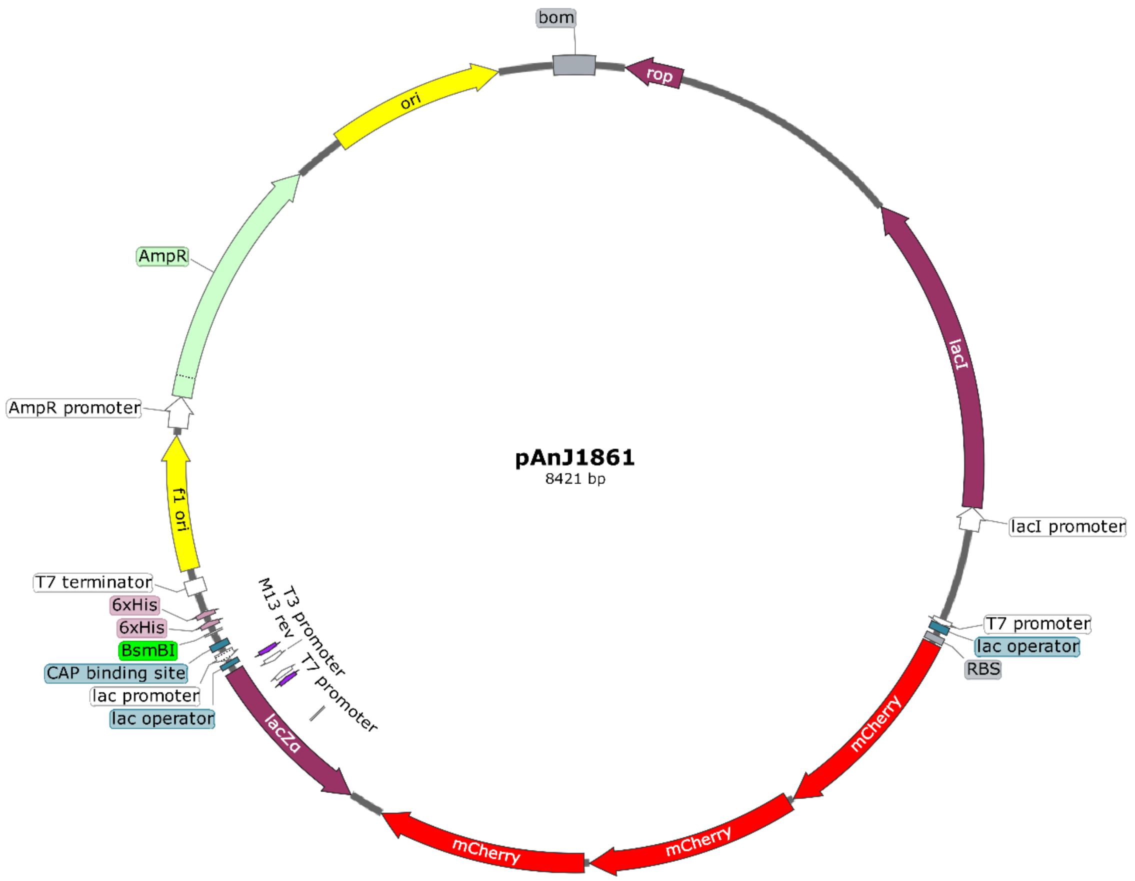


## pAnJ1861

Vector for bacterial expression of TALEs fused to three N-terminal mCherry and C-terminal 6xHis for protein purification.

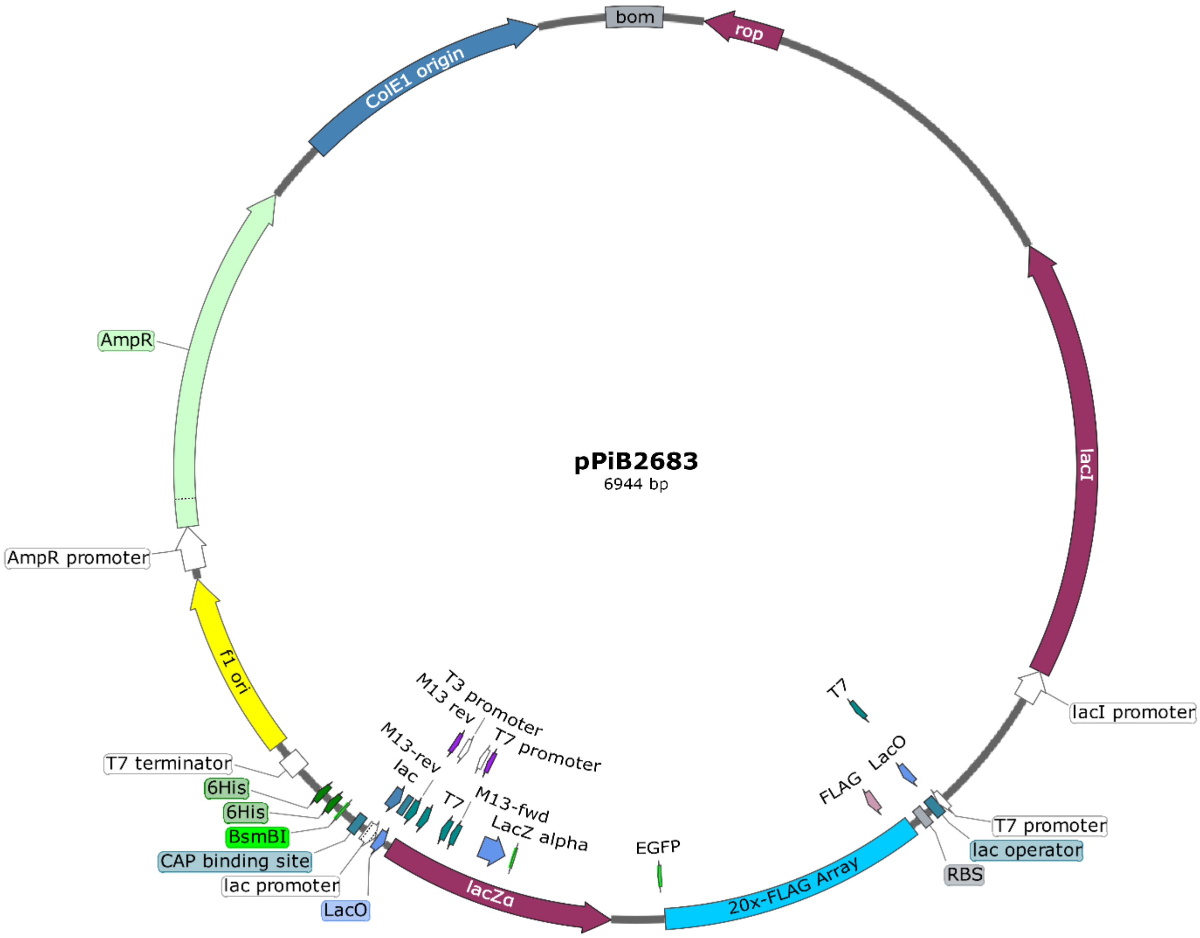


**pPiB2683**

Vector for bacterial expression of TALEs fused to 20 N-terminal FLAG tags and C-terminal 6xHis for protein purification.

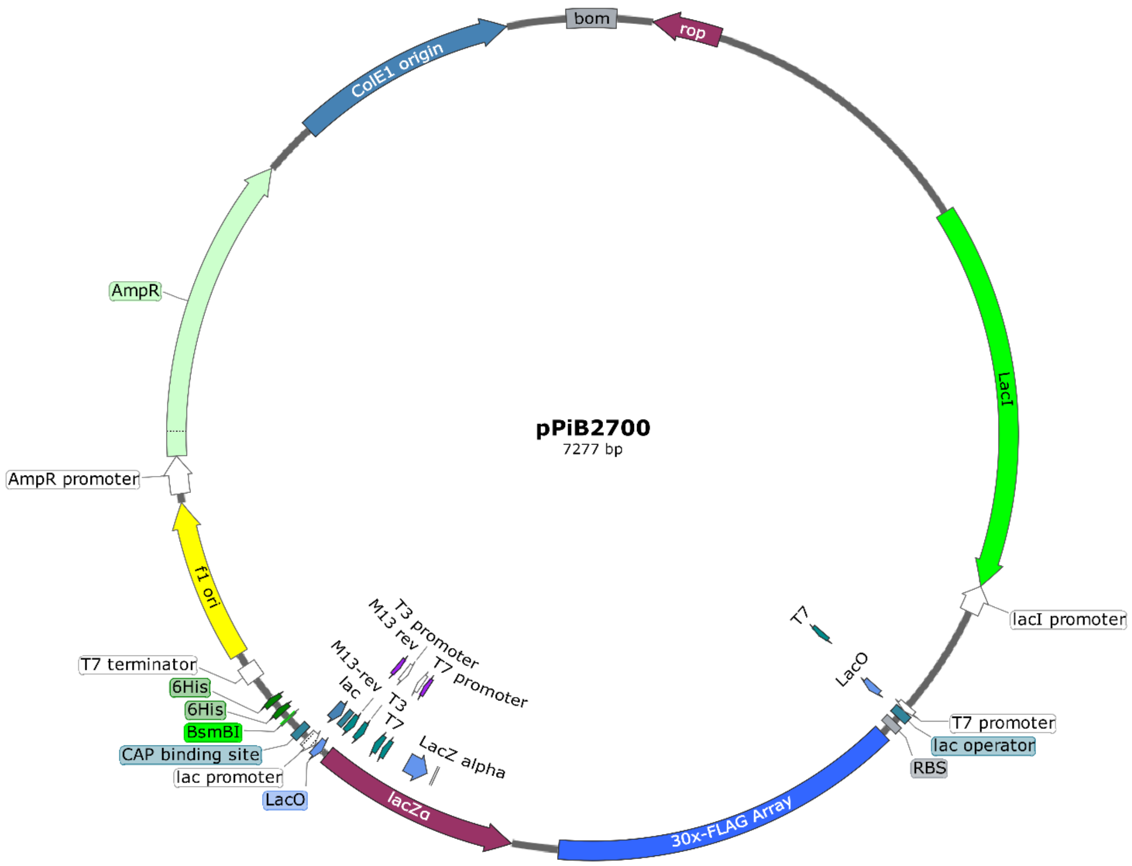


## pPiB2700

Vector for bacterial expression of TALEs fused to 30 N-terminal FLAG tags and C-terminal 6xHis for protein purification.

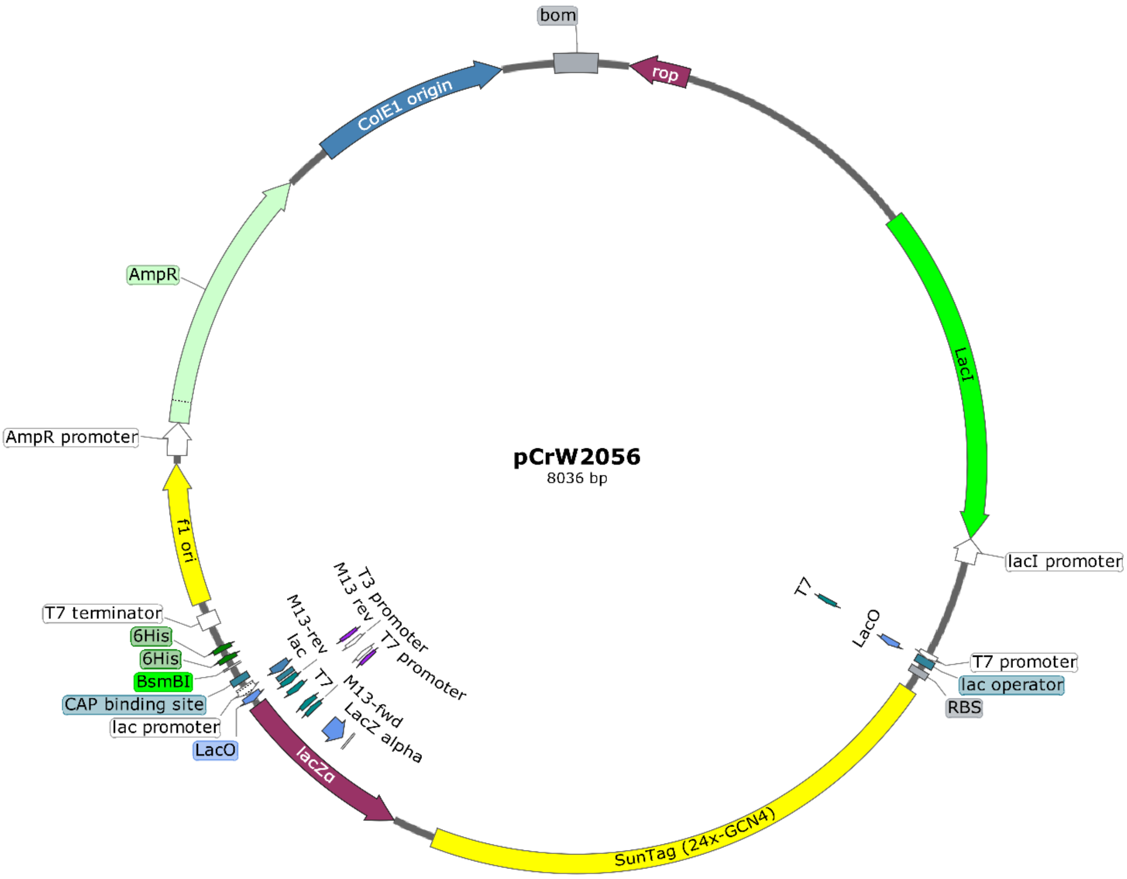


## pCrW2056

Vector for bacterial expression of TALEs fused to N-terminal SunTag (24x-GCN4) and C-terminal 6xHis for protein purification.

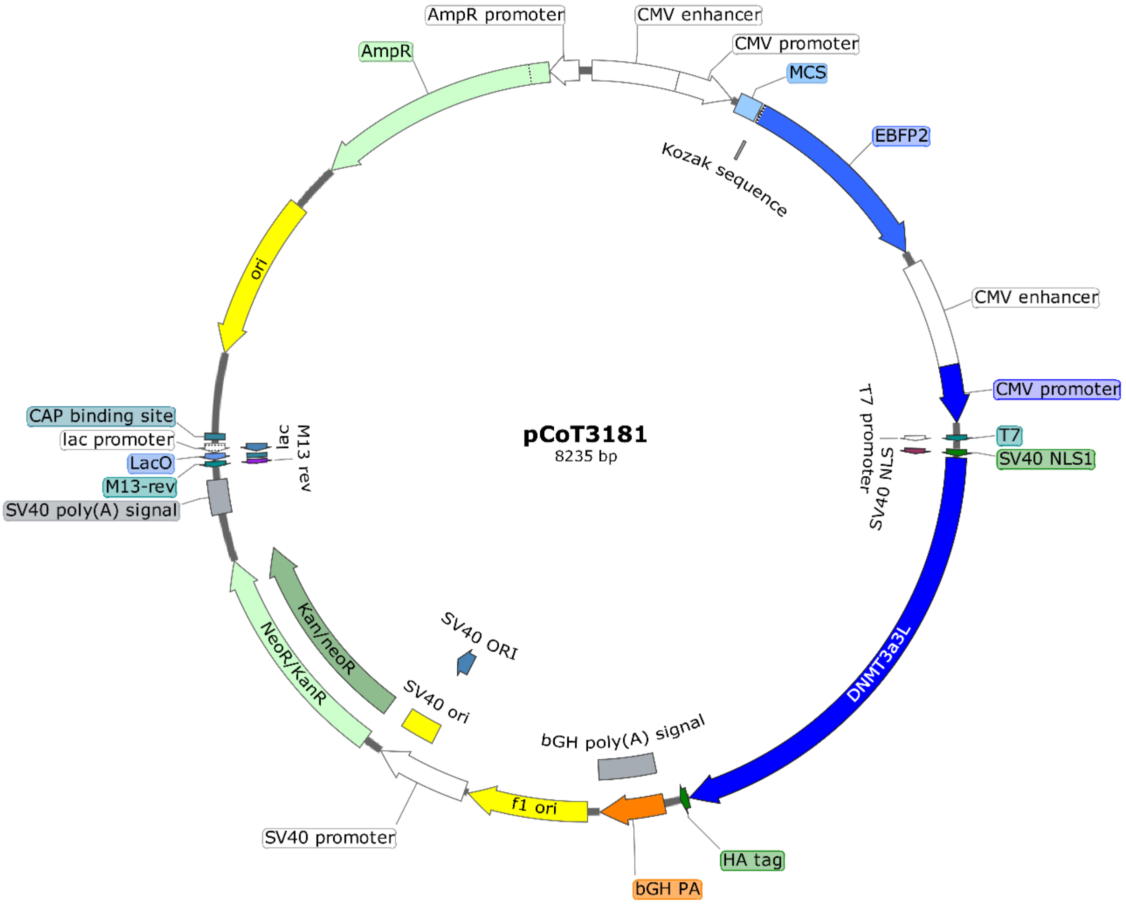


**pCoT3181**

Vector for mammalian expression of active DNMT3a3L for live-cell DNA methylation. As transfection control, the EBFP2 fluorophore stands under an independent promoter (CMV).

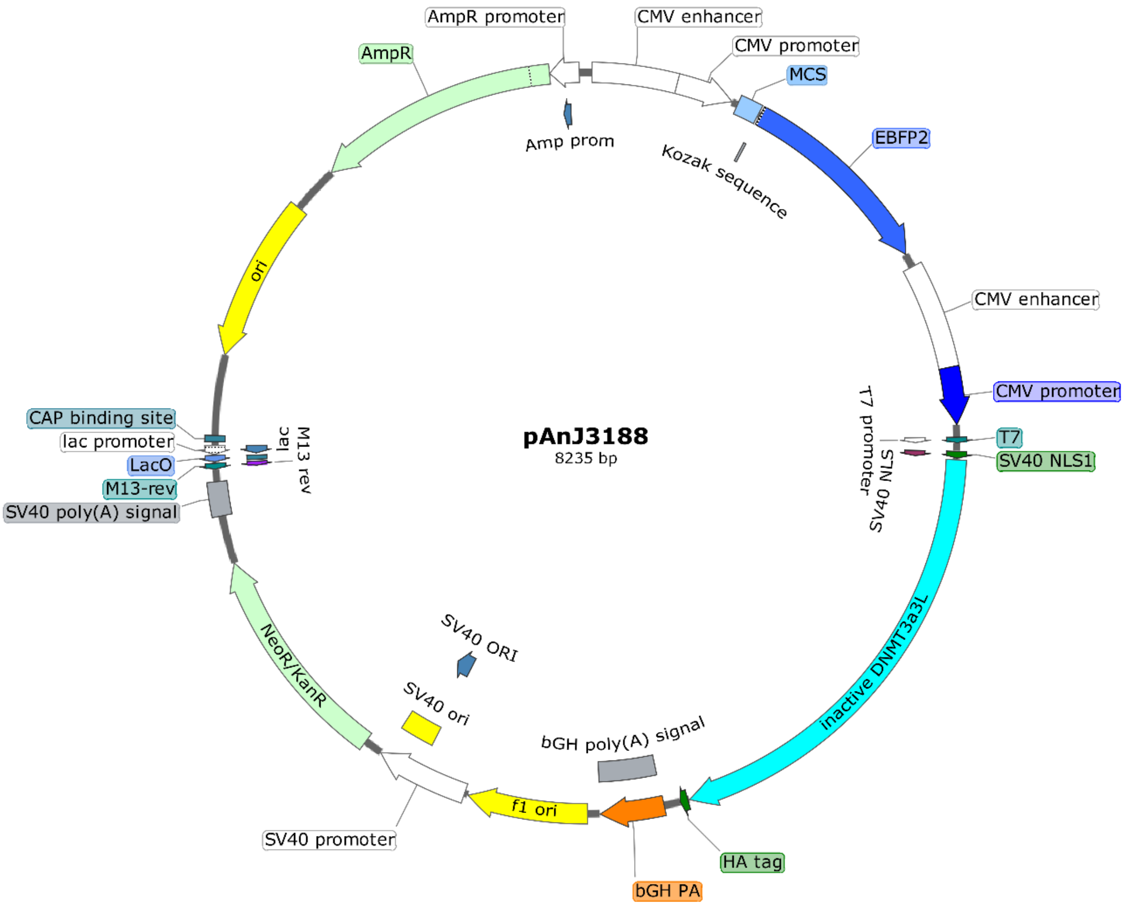


**pAnJ3188**

Vector for mammalian expression of inactive DNMT3a3L E756A for live-cell DNA methylation. As transfection control, the EBFP2 fluorophore stands under an independent promoter (CMV).
